# The Wnt non-canonical signaling modulates cabazitaxel sensitivity in prostate cancer cells

**DOI:** 10.1371/journal.pone.0234078

**Published:** 2020-06-02

**Authors:** Souad R. Sennoune, Thomas Nelius, Courtney Jarvis, Kevin Pruitt, Kameswara Rao Kottapalli, Stéphanie Filleur

**Affiliations:** 1 Department of Urology, Texas Tech University-Health Sciences Center, Lubbock, Texas, United States of America; 2 Department of Immunology and Molecular Microbiology, Texas Tech University-Health Sciences Center, Lubbock, Texas, United States of America; 3 American University of Antigua, College of Medicine, Antigua, West Indies; Southern Illinois University School of Medicine, UNITED STATES

## Abstract

**Background:**

Despite new drugs, metastatic prostate cancer remains fatal. Growing interest in the latest approved cabazitaxel taxane drug has markedly increased due to the survival benefits conferred when used at an earlier stage of the disease, its promising new therapeutic combination and formulation, and its differential toxicity. Still cabazitaxel’s mechanisms of resistance are poorly characterized. The goal of this study was thus to generate a new model of acquired resistance against cabazitaxel in order to unravel cabazitaxel’s resistance mechanisms.

**Methods:**

Du145 cells were cultured with increasing concentrations of cabazitaxel, docetaxel/ taxane control or placebo/age-matched control. Once resistance was reached, Epithelial-to-Mesenchymal Translation (EMT) was tested by cell morphology, cell migration, and E/M markers expression profile. Cell transcriptomics were determined by RNA sequencing; related pathways were identified using IPA, PANTHER or KEGG software. The Wnt pathway was analyzed by western blotting, pharmacological and knock-down studies.

**Results:**

While age-matched Du145 cells were sensitive to both taxane drugs, docetaxel-resistant cells were only resistant to docetaxel and cabazitaxel-resistant cells showed a partial cross-resistance to both drugs concomitant to EMT. Using RNA-sequencing, the Wnt non-canonical pathway was identified as exclusively activated in cabazitaxel resistant cells while the Wnt canonical pathway was restricted to docetaxel-resistant cells. Cabazitaxel-resistant cells showed a minimal crossover in the Wnt-pathway-related genes linked to docetaxel resistance validating our unique model of acquired resistance to cabazitaxel. Pharmacological and western blot studies confirmed these findings and suggest the implication of the Tyrosine kinase Ror2 receptor in cabazitaxel resistant cells. Variation in Ror2 expression level altered the sensitivity of prostate cancer cells to both drugs identifying a possible new target for taxane resistance.

**Conclusion:**

Our study represents the first demonstration that while Wnt pathway seems to play an important role in taxanes resistance, Wnt effectors responsible for taxane specificity remain un-identified prompting the need for more studies.

## Introduction

Prostate cancer (PCa) is the most commonly diagnosed cancer in men after skin cancer. For 2019, over 174,650 American men were expected to be diagnosed with PCa and more than 18% will die from the disease (American Cancer Society, Cancer Facts & Figures). Despite clinically controlled growth of localized PCa, metastatic/advanced PCa remains largely incurable, with rapid onset of lethality once the disease stages as castration-refractory metastatic PCa (mCRPCa).

Taxanes are microtubule-stabilizing drugs which block mitotic cell division leading to apoptosis [1]. Taxanes also act by inhibiting the androgen receptor (AR) signaling [2]. Docetaxel (Doc) with prednisone was the first approved regimen that showed survival benefits in mCRPCa patients [3,4]. While many patients respond initially, they ultimately develop resistance to the treatment. Cabazitaxel (Cab) was then later approved as second line taxane, based on its prolonged overall survival in Doc-resistant mCRPCa patients indicating activity that may help overcome resistance to prior taxanes [5]. Still patients inexorably die suggesting novel resistance [6]. While the interest towards Cab diminished because of its intervention at a late stage of the disease and the approval of new agents (Abiraterone Acetate, Enzalutamide…), important changes in patient treatment strategies recently emanated from several new clinical trials, which renewed the promise of this drug. STAMPEDE and CHAARTED clinical trials demonstrated that Doc in combination with Androgen Deprivation Therapy (ADT) was a more effective therapeutic alternative than ADT alone (> 13 months survival benefits) for metastatic androgen-sensitive PCa patients with high volume metastases [7,8]. Conversely, the FIRSTANA trial comparing Doc and Cab in mCRPCa, demonstrated that although both drugs did not differ in overall survival, Cab (25mg/m^2^) had a numerically higher tumor response than Doc and differed in its toxicity profile [9]. Importantly, these findings suggested for the first time that taxanes may be used at an earlier stage of the disease and that Cab could be an alternative to Doc (first line chemotherapy) in chemotherapy-naive patients. Besides, several studies support the utility and safety of Cab as either a second- or third-line agent after Doc; i.e.: **(a)** The re-challenge with Cab chemotherapy was shown as working in CRPCa patients treated with > 10 cycles of Doc, with an acceptable risk of adverse effects burden [10–12]; **(b)** Cab at a higher initial dose demonstrated longer survival duration after treatment than a lower initial dose for Doc-resistant mCRPCa patients [13]; **(c)** Cab/prednisone administered weekly to unfit mCRPCa patients appeared to be as effective as classical standard 3-week scheme (TROPIC study) but with significantly lower toxicities and better tolerance [14]; and **(d)** the combination of Cab and abiraterone was found to have a manageable safety profile and showed antitumor activity in patients previously treated with Doc and abiraterone [15]. Furthermore, the promising therapeutic findings on Cab benefits (as mono- or combined therapies) in other types of cancers such as cisplatin-resistant germ cell tumor cells [16], breast cancer [17,18], sorafenib-resistant hepatocellular carcinomas [19], mCRPCa [20,21], cytarabine-resistant leukemia [22], glioblastoma [23] or gliomas [24] impel for more studies to understand Cab modes of action. Furthermore, the current growing interest in developing Cab-carrying nanovehicles (liposomes, micelles, and nanoparticles) to reduce Cab’s side effects and increase its delivery and efficacy [17,18,25–34] urges again for more investigations on Cab.

Cab-resistance remains fairly new [35] with few single factors [Retinoblastoma tumor suppressor [36], SLCO1B3 transporter [37], FKBP7 chaperone protein, tubulins and the ABCB1 multidrug resistance protein] which have been linked to Cab sensitivity [38–43]. In contrast, enhancement of MAPK/ERK, PI3K/AKT or CCL2-CCR2 signaling was detected in Cab-resistant mCRPCa cell lines [44,45] connecting for the first time specific pathways to Cab sensitivity. Still more work is required to identify signaling pathways involved in CabR. To fill this gap, we have thus established a cellular experimental model of acquired resistance against Cab. As comparison and age control, DocR and age-matched cell lines were respectively established. Using RNA-sequencing, we identified the Wnt non-canonical pathway as exclusively activated in CabR cells while the Wnt/β-catenin canonical pathway seemed to be restricted to the Doc counterpart cells. Accordingly, pharmacological and western blot studies confirmed these findings and suggest the implication of the Tyrosine kinase Ror2 receptor in Cab resistance. Surprisingly, variation in Ror2 expression level altered the sensitivity of PCa cells to both Doc and Cab suggesting that the Wnt pathway may represent a new target for resistance to taxanes in PCa cells.

## Materials and methods

### Cells, cell culture, drug treatments and reagents

The human PCa cell lines, Du145, RWPE-1, and RWPE-2 were purchased from the American Type Culture Collection (ATCC, Manassas, VA). Du145 cells were maintained in EMEM (ATCC) medium supplemented with 10% Fetal Bovine Serum (FBS, Hyclone, Logan, UT) and 1% penicillin/streptomycin (HyClone) at 37°C in 5% CO_2_. The RWPE-1 and RWPE-2 cells were maintained in Keratinocyte-Serum Free Media supplemented with human recombinant Epithelial Growth Factor and Bovine Pituitary Extract (all from Gibco, ThermoFischer). To study the effect of Doc and Cab treatment on RWPE-1 and RWPE-2 cells, the cells were seeded at 1x10^4^ per well in 24 well plate, then the following day, RWPE-1 and RWPE-2 cells were treated with Doc or Cab with different doses (6.25, 12.5, 25, 50, 100 and 200 nM) and incubated for 24hrs. The treated cells were then fixed with 2% glutaraldehyde for 10 minutes, washed with PBS and then stained with crystal violet for 40 minutes and then the plates were washed with water and air dry overnight. The absorbance of crystal violet was read at 590 nm using the Quad4 monochromators plate reader (Tecan Infinite M1000 Pro multi detection).

**Fig 1:**
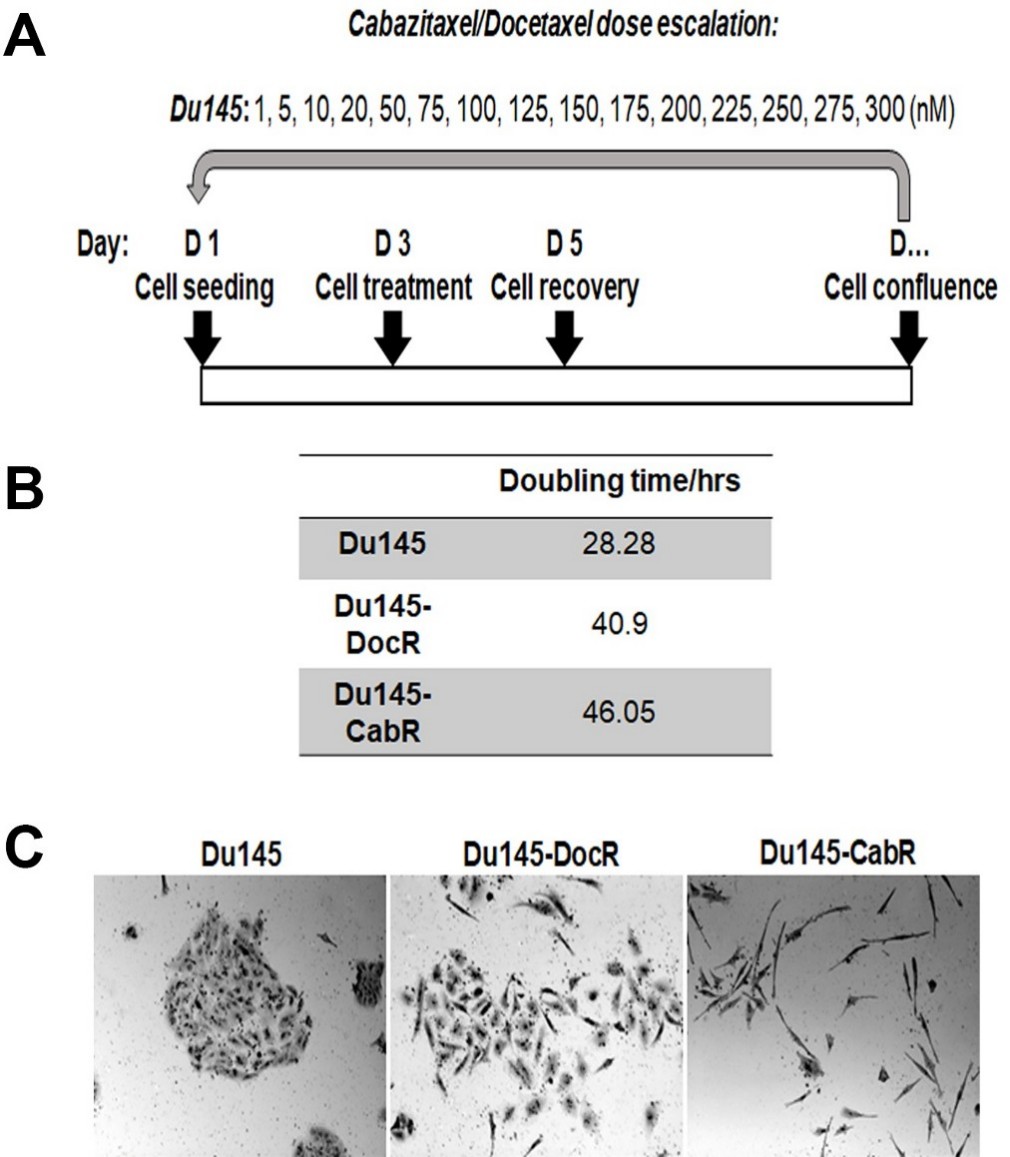
Establishment of taxanes resistant cell lines **(A)** Treatment scheme for the Du145 cells. **(B)** Doubling times of the age-matched, DocR and CabR derivative cell lines. **(C)** Cell morphology as observed under a light microscopy using a 20x objective. Cells doubling time was calculated over a 9 consecutive day period using the formula N(t) = C2^t/d where N(t) = number of objects at time t, d = Doubling period, C = initial number of objects and t = time [46].

For the development of taxane-resistant PCa cells, Du145 cells were seeded at 3 x 10^4^ cells/ml in T-25 cell culture flasks and allowed to grow for 48 hours before being treated with dose escalation (Fig 1) of Doc, Cab or DMSO using 72 hour exposure time. Dose escalation was pursued unless cells became insensitive to the drug. Resistant cells were then maintained in complete medium with the drug (EC_50_). Age-matched cells were grown alongside as an age control. The resistant cell lines were established in a 6-month time period. Resistant/age-matched cell lines were then authenticated by the ATCC and designated as age-matched, Doc resistant (DocR) and Cab resistant (CabR). Cells doubling time (Fig 1C) was calculated over a 9 consecutive day period using the formula N(t) = C2^t/d where N(t) = number of objects at time t, d = Doubling period, C = initial number of objects and t = time [46]. EC_50_ values were calculated as previously described (55). Doc and Cab drugs were purchased from Fluka (Mexico City, Mexico) and Medchem Express (Monmouth Junction, NJ). Gö6983, SP600125, and KN93 inhibitors were all from Sigma Aldrich (St. Louis, MO).

### Cell morphology

The cell morphology was imaged using an Olympus BX50 confocal microscope and crystal violet staining as well as quantified using an Amnis ImageStream MarK II flow cytometer. For the quantification data, the cells were fixed in 2% glutaraldehyde (10 min) then scraped and initially gated for single cells before recording 10,000 events. Cell morphology was analyzed using the IDEAS software (Image Data Exploration and Analysis Software, Version 4.0, Amnis Corporation, Seattle, WA) based on three parameters: circularity, aspect ratio and elongatedness [47]. Circularity describes how the degree of the mask’s deviation from a circle. Aspect ratio is the ratio of the minor axis divided by the major axis. Elongatedness describes the ratio of the height/width, which use the bounding box feature. F-actin and tubulin immunostaining was also performed. Cells seeded onto glass coverslips were fixed with 4% paraformaldehyde in PBS for 15 minutes before being quenched with PBS-NH_4_Cl for 5 minutes. Cells were then permeabilized with PBS-Triton X-100 0.2% for 5 min and blocked in 5% PBS-BSA for 60 minutes. Cells were incubated with tubulin antibody (Abcam) at a dilution of 1/500 (in 1% PBS-BSA), overnight at 4°C. Cells were washed several times with PBS and then incubated with secondary antibody Alexa Fluor 568 anti-rat (Abcam) at a dilution 1/1000, and Alexa Fluor 488 phalloidin to label F-actin at a dilution of 1/1000, for 60 minutes at room temperature. The glass slides were then mounted using Prolong Gold antifade reagent with DAPI (ThermoFisher Scientific) and cured for 24 hours before being imaged. Imaging was preformed using a Nikon T1-E 200 microscope (63X objective, Z-stack).

### TUNEL/apoptosis assay

Du145 cells were seeded in 6 well dish on glass coverslips at 1 x 10^5^ cells/well and treated with 10nM of Doc or Cab for 24 hours. Cells were then fixed, permeabilized and washed before being stained with the ApopTag Fluorescein In Situ Apoptosis Detection Kit (EMD Millipore, Temecula, CA) following the manufacturer’s recommended protocol. Cells (> 1,000 cells per treatment condition) were imaged using a Zeiss 200M Axiovert microscope (20X objective). Cells presenting positive FITC staining with signs of cell death within the nucleus (chromatin condensation and chromosomal DNA fragmentation visualized by DAPI staining) were counted as apoptotic. For the inhibitors study, cells were treated with Gö6983 (PKC inhibitor, 1μM, 2hrs), SP600125 (JNK inhibitor, 30μM, 2hrs), and KN93 (CamKII/IV inhibitor, 10μM, 4hrs) alone or in combination followed by either 20nM Doc or Cab for 24 hours.

### Clone formation assay

Du145 cells were seeded at 5 x 10^4^/well in 6-well dishes and allowed to grow for 48 hours. Cells were then treated in complete medium with 50nM of either Doc or Cab [48]. After 72 hours, drugs were replaced with complete medium; culture medium was refreshed every 3 days during the whole experiment. After 30 days, cells were fixed in a 2% glutaraldehyde solution for 10 minutes and stained with 0.1% crystal violet (dissolved in water and filtered) for 40 minutes before being washed and air dried. Total colonies in each well were then counted. Each treatment was carried out in triplicate.

### *In vitro* migration assay

was performed as we described previously [49] with few minor modifications. 1.5 x 10^6^ cells/ml were seeded on top of the gelatinized microporous membrane (8μm pore size, 6.5 mm Corning insert). After two hours, serum-free media was added to the bottom part of the well and incubated at 37°C overnight. Migrated cells were then fixed and stained using the Diff-Quick staining kit (Dade Behring, Deerfield, IL) and counted in 10 high-powered fields (100X).

### RNA sequencing

RNA was isolated from Du145 age-matched, CabR and CabR re-challenged (to access Cab-induced changes in gene expression in potential drug resistant pathways) cells using the GenElute™ Total RNA Purification Kit (Sigma-Aldrich, St. Louis, MO). RNA concentration, quality and integrity were verified on a Qubit (Life Technologies, Carlsbad, CA) and Agilent TapeStation 2200 (Agilent Technologies, Santa Clara, CA). RNA-Seq libraries from three replicates per cell line and treatment condition were constructed using the Illumina TrueSeq Stranded mRNA library Prep kit on the NeoPrep system (Illumina, San Diego, CA). Quality of the libraries was then checked using the Agilent Tapestation 2200 and quantified using the Qubit. Pooled libraries were sequenced using an Illumina HiSeq 2500 sequencer (Illumina). Sequencing run data containing base call information was de-multiplexed in Base-Space Sequence Hub cloud-based genomics computing environment for next-generation sequencing (NGS) data management and analysis tool. As we previously described [50], the resulting fastq.gz files were decompressed using 7-zip software. Fastq files were then generated for read 1 and read 2 for each sample. FastQC (Babraham Bioinformatics, Cambridgeshire, England, UK) was used to check Phred quality score (Q score). For raw-read alignment and differential gene expression analysis fastq files were imported into QSeq Version 14.0 software (DNASTAR, Madison, WI) and aligned to the Homo sapiens GRCh38.p7 (NCBI) with the Qseq software (DNASTAR). Reads assigned per kilobase of target per million mapped reads (RPKM) normalization was done prior to quantification of Differentially Expressed Genes (DEGs) by QSeq software. Student's two-tailed unpaired t-test with Benjamin Hochberg FDR correction was used to compare the means of gene expression values. Genes were identified as statistically significant and differentially expressed with a threshold for false discovery rate <0.01 and a fold-change ≥2.0. DEG with annotations, Log2 fold-change, and p-value were exported for input into Ingenuity Pathway Analysis Software (IPA, QIAGEN Redwood City) tool core analysis using standard settings with duplicates resolved. DEG lists were loaded into PANTHER Classification System for gene ontology [51,52]. DEGs including Log2 fold-change were saved as a tab delimited text file. The file was uploaded, and heat maps were generated using Gene-E (Broad Institute, https://software.broadinstitute.org/GENE-E/).

### Quantitative RT-PCR

Total RNAs were extracted (RNeasy extraction kit, Qiagen, Valencia, CA) and 2 μg of RNA were converted into cDNA using the Thermo Scientific VersoTM cDNA synthesis kit (Thermo Scientific, Grand Island, NY) [53]. cDNA was then amplified (FastStart Universel SYBR Master Mix, Roche) in an MyiQ amplifier (BioRad, Hercules, CA). The program used was 1 cycle of heating for 10’ at 95°C; followed subsequently by 40 cycles of denaturating for 20” at 95°C, and annealing/extension for 1’ at 60°C. Primers for human S15 and Ror2 were purchased from Qiagen. The housekeeping gene S15 was used as reference. Cycle threshold (CT) values were obtained by automated threshold analysis with MyiQ version 1.0 software and the fold change for each gene calculated using the ΔΔCt method. Each sample was tested in triplicate. All the qRT-PCR products were run on a 2% agarose gel and showed amplicons of correct sizes.

### Western blotting

Total proteins were extracted using RIPA buffer [25mM Tris, pH 7–8, 150mM NaCl, 0.1% SDS, 0.5% sodium deoxycholate, and 1% NP-40], separated by 10% SDS–PAGE and transferred onto Immobilon-P transfer membrane. After blocking in T-TBS (20mM Tris, 150mM NaCl and 0.1% Tween 20, pH 7.5) containing 5% nonfat dry milk, the membranes were incubated with primary antibodies [(E-cadherin (cell Signaling, Danvers, MA), Ror2, β-catenin, Ror1, Vimentin, CK19, PKC, JNK1-3, and CamKII/IV (Santa Cruz Biotechnology, Dallas, TX)]. The membranes were then incubated with a horseradish peroxidase-conjugated secondary antibody. The signal was revealed using the Prime Western Blotting kit (Amersham, Malborough, MA). After stripping, the membrane was re-probed for β-actin to assess loading.

### Immunofluorescence

Cells seeded onto glass coverslips were fixed with 4% paraformaldehyde in PBS for 15 minutes before being quenched with PBS-NH_4_Cl for 5 minutes. Cells were then permeabilized with PBS-Triton X-100 0.2% for 5 min and blocked in 5% PBS-BSA for 60 minutes. Then, the cells were incubated with E-cadherin antibody (Cell Signaling, Danvers, MA) at a dilution of 1/200 in 1% PBS-BSA, overnight at 4°C. Cells were washed several times with PBS and then incubated with secondary antibody Alexa Fluor 568 anti-rabbit (Thermo Fisher Scientific, Grand Island, NY) at a dilution 1/1000 for 60 minutes at room temperature. After several washes with PBS, the glass slides were then mounted using Prolong Gold antifade reagent with DAPI (Thermo Fisher Scientific) and cured for 24 hours before being imaged. Imaging was preformed using a Nikon T1-E 200 microscope (63X objective, Z-stack). The images were analyzed with NIS software. Data are represented as a full, non-cropped original image.

### Gene knock-down

For Ror2 siRNA, the siGenome SMARTpool human Ror2 and the siGENOME non-targeting siRNA (NT siRNA) control were designed and synthesized by Dharmacon (Lafayette, CO). 70% confluent cells were transfected with Ror2 or NT siRNA (25nM) using the DharmaFECT Transfection Reagent following the recommended protocol. After 72–96 hours, cells were washed in complete media and treated with taxanes or carrier control. Transfection was verified using the fluorescent siGlo (fluorescent RNA duplex used as positive control for the transfection of siRNA, designed and synthesized by Dharmacon).

### Statistical analysis

Significant differences were determined by using an ANOVA procedure with Holm-Sidak's test (normal distribution) or the Kruskal-Wallis ANOVA with Dunn's test (nonparametric distribution; SigmaStat). Student's two-tailed unpaired t-test with Benjamin Hochberg FDR correction was used to compare the means of gene expression/protein values. Differential expression detection was performed using QSeq software. Standalone R program R-GSEA (http://www.broadinstitute.org/gsea/ downloads.jsp) was used for pathway analysis. Data analyses were performed using SAS (version 9.13; SAS Institute) or R (http://www.R-project.org). P ≤ 0.05 was considered statistically significant.

## Results

### Generation of cabazitaxel-resistant cells

**Fig 2:**
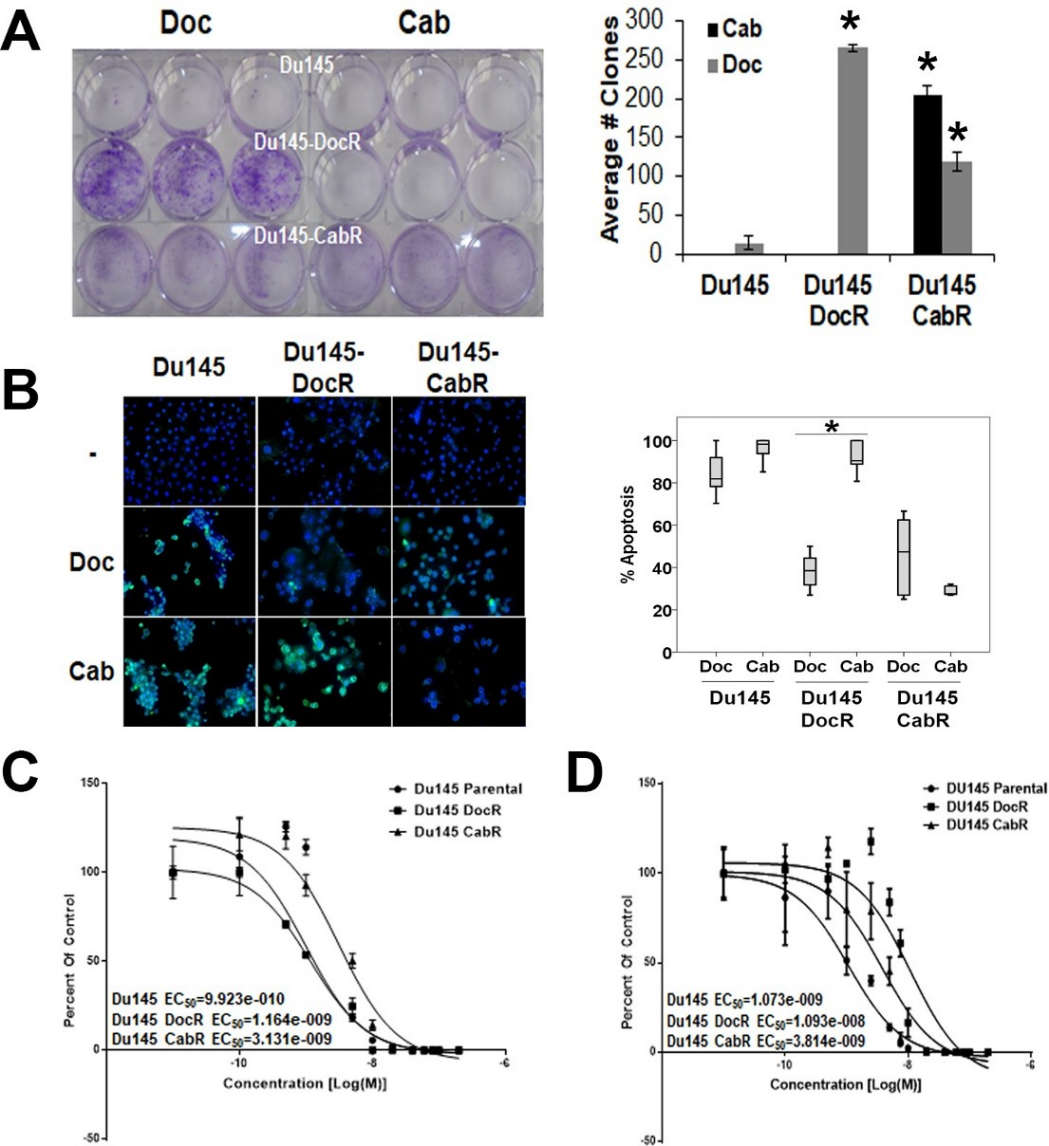
*In vitro* validation of the resistance against taxanes in Du145 cells. **(A) Left panel:** representative pictures showing the formation of clones by Du145 age-matched, DocR and CabR cells under 50nM Doc or Cab. **Right panel:** Quantification of clone formation counted 30 days after ending the treatment. Error bars: mean ± SD from triplicates. *: P < 0.05 showing statistical significance. **(B) Left panel:** Representative pictures of apoptosis in Du145 age matched, DocR and CabR resistant cells using TUNEL assay. Cuts in DNA (Green) and nucleus staining with DAPI (Blue) are shown. **Right panel:** quantification of apoptosis (Boxplot graphs showing the median, inter-quartile range, upper and lower quartiles, and whiskers) measured from more than 300 cells per condition treatment. The experiment was repeated three times. Statistical analysis was performed using one-way ANOVA on Ranks, Kruskal-Wallis Analysis, followed by Dunn’s post-hoc test. *P<0.05). **(C-D)** Growth curves of age-matched, DocR and CabR cells under increasing concentration of Doc **(C)** or Cab **(D).** After 24 hours treatment, cells were fixed, stained using crystal violet solution, rinsed and air dried. Alive cells [cells that do not present cell death signs within the nucleus (chromatin condensation and chromosomal DNA fragmentation visualized by DAPI staining)] were then counted in each well. Each treatment condition was tested in triplicate; each experiment repeated at least three times. Quantification was done from three different experiments and data are expressed as mean ± SD.

To develop a cell experimental model for acquired resistance to Cab, we used the human CRPCa Du145 cell line that we exposed to dose escalation of Doc, Cab or placebo (age-matched control; Fig 1A). Once cells became unresponsive to the drugs (Fig 1B and 1C), doubling times for the age-matched, DocR, and CabR were calculated. Resistance was then validated by clone formation and *in vitro* TUNEL apoptosis assays (Fig 2A and 2B). In the two assays, age-matched parental cells showed sensitivity to both drugs. DocR cells were only resistant to Doc. In contrast, CabR cells formed twice as many colonies in Cab than Doc suggesting a partial cross-resistance (Fig 2A). EC_50_ values for the age-matched, DocR, and CabR from cells treated with either Cab or Doc are shown in Fig 2C and 2D, respectively.

### Cabazitaxel resistant Du145 cells underwent epithelial-mesenchymal transition

**Fig 3:**
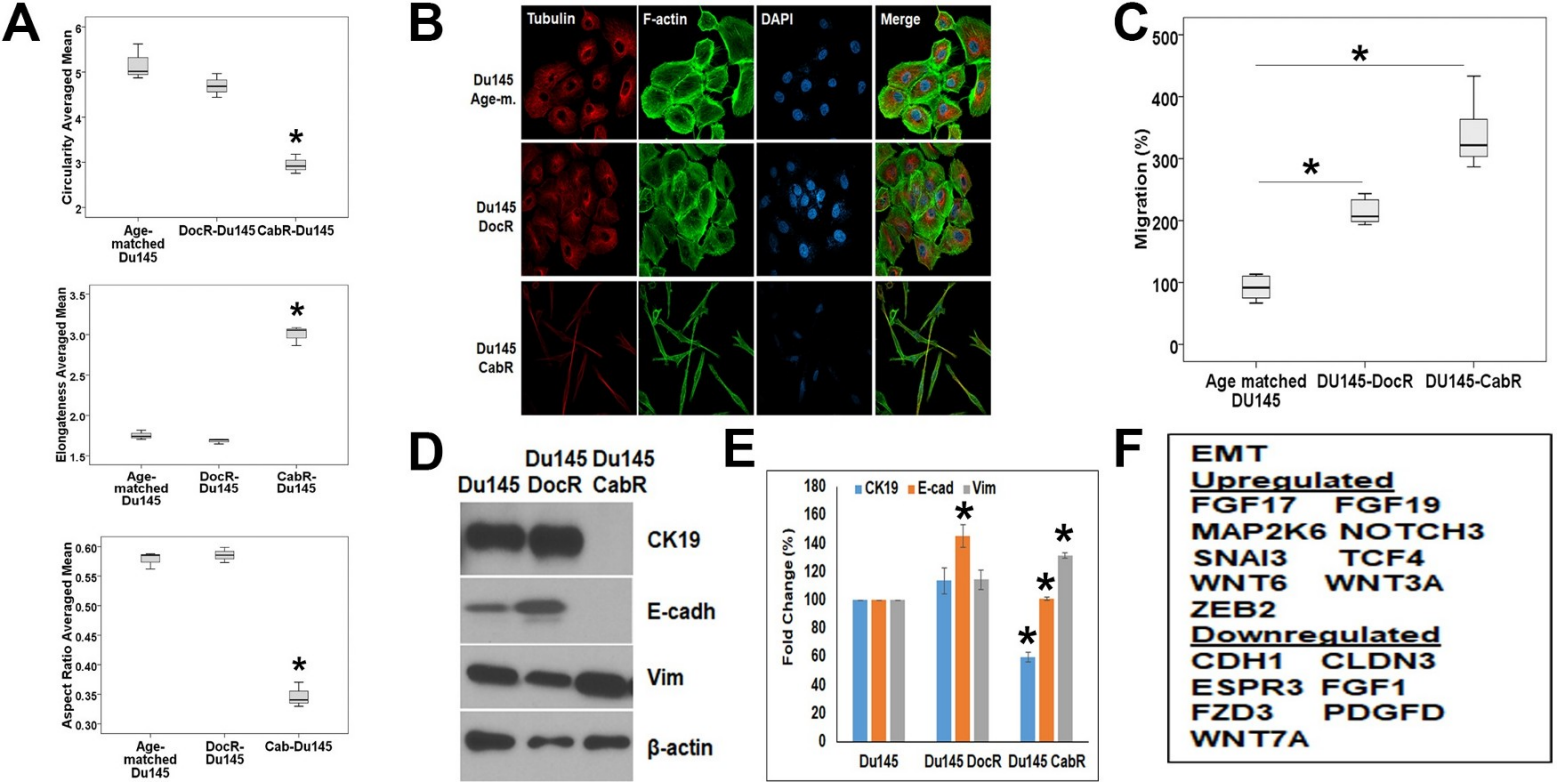
Genotypic and phenotypic characterization of the taxanes resistant Du145 cells **(A)** Cell morphological study looking at the cell circularity (Top), elongatedness (Middle), and aspect ratio (Bottom). Statistical analyses were performed using the Welch and Brown-Forsythe tests followed by the Games-Howell test, *p < 0.05. **(B)** Cell morphology analyses showing the distribution of tubulin (Red staining) and F-actin (Green staining) in Du145 age-matched, DocR, and CabR. Nuclei are visualized by DAPI staining (Blue). **(C)** Migration of the age-matched, DocR and CabR cells using the Inverted Boyden Chamber *in vitro* assay. Migration of the age-matched cells was used as the 100% migration control. Each cell line was tested in quadruplicate and the experiments were repeated three times. **(D)** Protein expression level of Epithelial (Cytokeratin 19 and E-cadherin) and Mesenchymal (Vimentin) specific markers. Beta-actin was used as a protein loading control. **(E)** Protein expression quantification obtained from three separate western blotting experiments. Decreased E-cadherin and CK19, and increased vimentin proteins in CabR cells when compared to the age-matched or DocR cells were all found statistically significant. Statistical analyses were done using a t-test. **(F)** mRNA expression profile of Epithelial and Mesenchymal specific markers.

To investigate and quantify the morphological changes experienced during the generation of taxane-resistant Du145 cell lines (Fig 1C), cell morphology assays were performed. Flow cytometry analysis revealed that CabR cells had a significantly reduced circularity and aspect ratio compared to age-matched and DocR Du145 cells (Fig 3A). Increased cellular elongation was also observed for CabR cells. Similar results were found when F-actin and tubulin were co-immuno-stained (Fig 3B) suggesting an Epithelial-Mesenchymal Transition (EMT) phenomenon. Accordingly, taxane-resistant cells migrated more than the age-matched parental cell line with CabR cells migrating more than the DocR Du145 line (Fig 3C). The expression of epithelial and mesenchymal specific markers were then measured using western blotting. While Cytokeratin 19 (CK19) completely disappeared in CabR cells, E-cadherin protein was respectively expressed at a low level, up- and down-regulated in age matched, DocR, and CabR cells. These findings were corroborated by immunofluorescence (S1 Fig). We found that E-cadherin staining was stronger in DocR compared to age-matched parental cells and disappeared in CabR cells. Furthermore, in addition to its subcellular location, E-cadherin was observed in the nucleus and the staining was stronger in DocR cells. Accordingly, Vimentin protein expression was significantly up-regulated in CabR compared to age-matched Du145 and DocR cells validating EMT (Fig 3D and 3E) as previously described in breast cancer [35]. Fig 3F shows genes such as *SNAI3*, *TCF4*, *WNT6*, *WNT3A*, and *ZEB3* that were up-regulated in Du145-CabR cells. In contrast, *CDH1*, *CLDN3*, *ESPR3*, *FGF1*, *FZD3*, *PDGFD* genes were down-regulated.

### *In vitro* transcriptomics characterization of CabR Du145 cells

**Fig 4:**
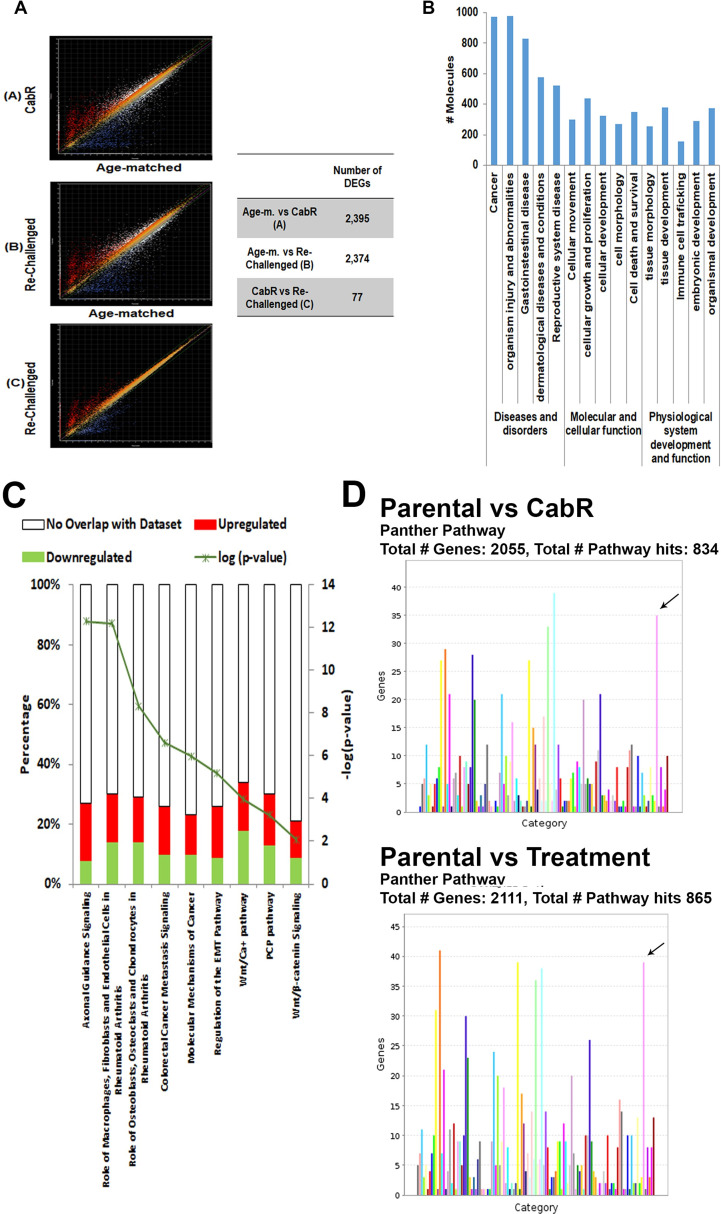
RNA-sequencing analysis. **(A)** Scatter plot diagrams were constructed to represent the numbers of DEGs (Differentially Expressed Genes) among the Du145 age-matched, CabR, and re-challenged CabR cell lines. Genes significantly upregulated and downregulated (> two fold change) are shown. **Right Table:** DEGs quantification analysis. **(B)** IPA (Ingenuity Pathway Analysis-Qiagen) was used to determine altered pathways in CabR cells. **(C)** Pathway analysis in CabR cells. The top canonical pathways (n = 45) included axonal guidance signaling, role of macrophages, fibroblasts and endothelial cells in Rheumatoid Arthritis, Hepatic Fibrosis/Hepatic Stellate cell activation, role of osteoblasts, osteoclasts and chondrocytes in Rheumatoid Arthritis, and colorectal cancer metastasis signaling. **(D)** DEG analysis using the PANTHER Classification System for gene ontology and identifying the Wnt signaling pathway (indicated by the black arrow) as the second most represented pathway present in our dataset (Age-matched vs CabR; age-matched vs Cab re-challenge).

To characterize the molecular mechanisms involved in CabR, we performed a whole transcriptomics analysis on the age-matched and CabR cells. Additionally, CabR cells shortly re-challenged with the drugs (50nM for 24hrs) were integrated to the analysis to evaluate any effect of a short-term exposure to the drug on potentially identified signaling pathways. After RNA-sequencing, quality filtered reads were mapped on the human genome Homo sapiens GRCh38.p7 (NCBI) with the Qseq software. Scatterplots of pairwise comparisons of differentially expressed genes (DEGs, Fig 4A) between age-matched and CabR (**A**), age-matched and re-challenged (**B**) and CabR and re-challenged (**C**) cells were then created. Each comparison was limited to a 99% confidence interval and a 2-fold expression change. Our analysis showed that the age-matched/CabR and age-matched/re-challenged comparisons exhibited the largest differential expression with 2,395 and 2,374 DEGs, respectively, demonstrating the highest level of dissimilarity. In contrast, CabR/re-challenged generated only 77 DEGs (Fig 4A) showing high level of similarity.

IPA (Ingenuity Pathway Analysis-Qiagen) was then used to determine altered pathways in CabR cells (Fig 4B). The major diseases and disorders functions were cancer, organismal injury and abnormalities, and gastrointestinal disease. The main molecular and cellular functions included cellular movement, growth and proliferation, cell morphology, and cellular development (Fig 4B). The top canonical pathways (n = 45) included axonal guidance signaling, role of macrophages, fibroblasts and endothelial cells in Rheumatoid Arthritis, Hepatic Fibrosis/Hepatic Stellate cell activation, role of osteoblasts, osteoclasts and chondrocytes in Rheumatoid Arthritis, and colorectal cancer metastasis signaling (Fig 4C). Importantly, all of these pathways have been linked to Wnt signaling, one of the top 5 signaling pathways that we have also identified using the GoPanther analysis [Fig 4D (black arrows), and S2 Fig]) [54–56]. In addition to Wnt-related genes, genes previously identified in taxane resistance were also present in our data set (PDGFD, ABCG2, ABCC2, and SLCO1B3) [57–62]. Similarly to Hongo’s study, we also identified the MAPKinase pathway as one of the top pathway of our CabR-Du145 cell data set [44]. In contrast, CabR cells showed a minimal crossover in genes linked to DocR (S3 Fig) thus validating our resistance model, but also demonstrating the unicity of our acquired resistance model to Cab.

### Cabazitaxel resistant cells showed increased gene expression in the Wnt non-canonical pathway

**Fig 5:**
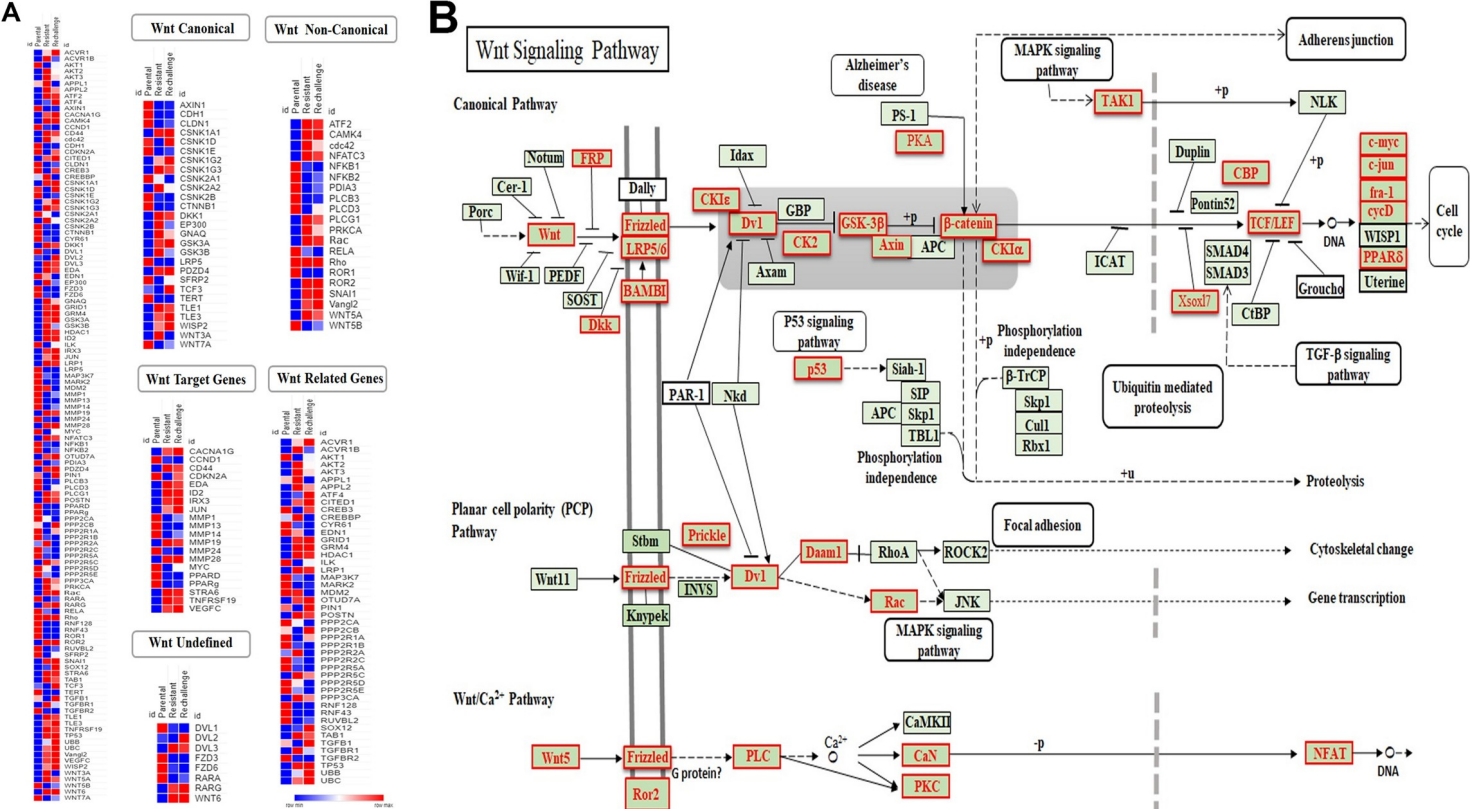
Major representation of Wnt non-canonical genes in CabR cells. **(A)** Heat map (GENE-E software) showing the expression profiles of specific effectors of the Wnt pathways in age-matched (parental), and CabR (resistant) and re-challenged Du145 cells. **(B)** Wnt pathways generated using KEGG (Kyoto Encyclopedia of Genes and Genomes) software. Genes found differentially expressed in our RNA-Seq data set are highlighted in red.

**Fig 6:**
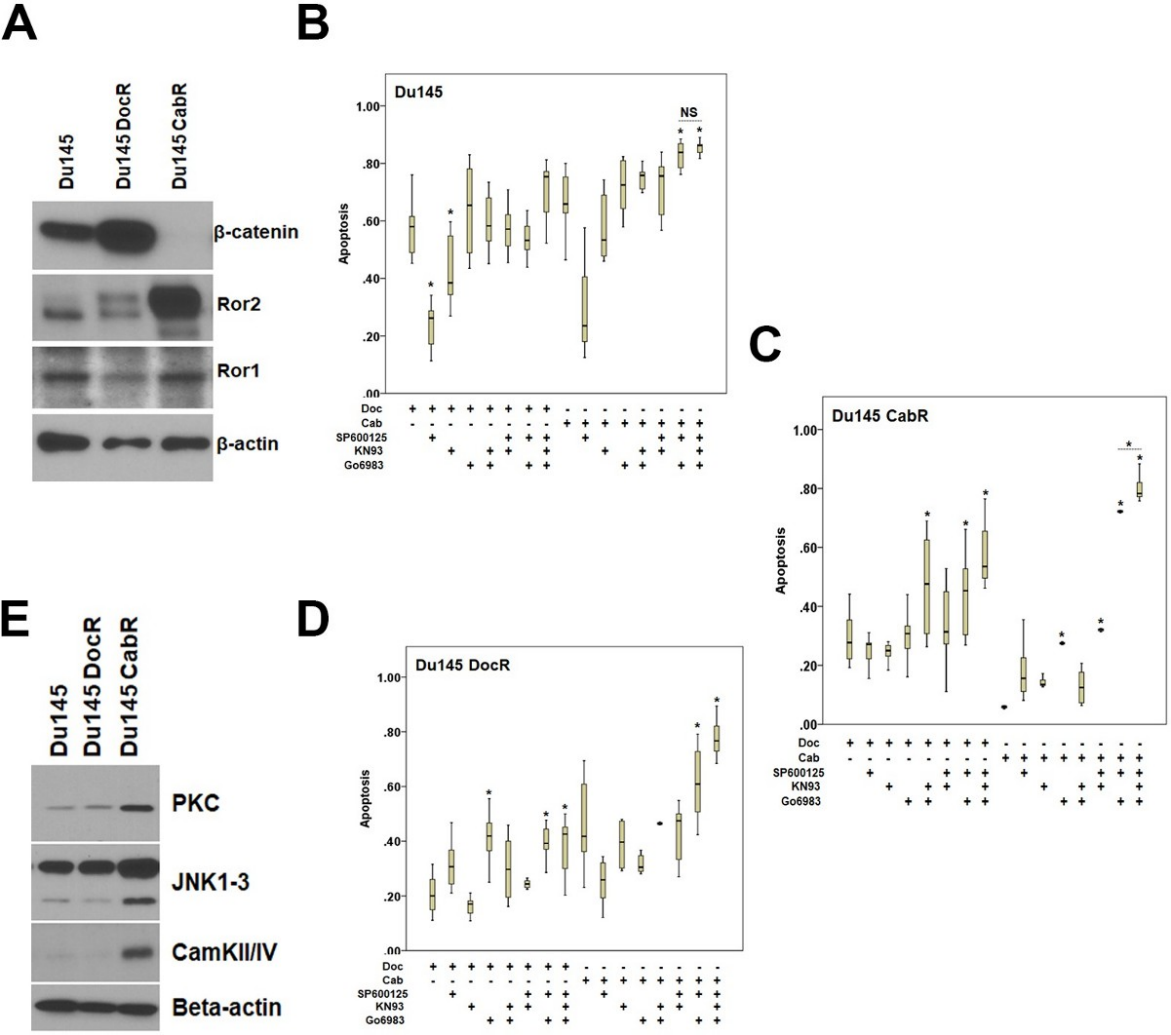
Role of the Wnt pathway in taxane resistance. **(A)** Representative expression levels of the β-catenin, Ror1, and Ror2 proteins in age-matched and taxane resistant Du145 cells. In these experiments, three independent protein cell extracts were tested. **(B-D**) *In vitro* pharmacological study. TUNEL apoptosis assay was performed in age-matched **(B)** and taxanes-resistant **(C-D)** Du145 cells treated with Gö6983 (PKC inhibitor, 1μM, 2hrs), SP600125 (JNK inhibitor, 30μM, 2hrs), and KN93 (CamKII/IV inhibitor, 10 μM, 4hrs) combinations followed by taxanes (50nM, 24hrs). **(E)** Expression level of PKC, JNK and CamkII/IV proteins showing a specific up-regulation of all three kinases in CabR when compared to age-matched and DocR cells. Beta-actin was used to control the loading.

To characterize the Wnt signaling pathway associated with CabR, heat-maps were generated using Gene-E (Broad Institute, https://software.broadinstitute.org/GENE-E, Fig 5A). 120 DEGs associated with the Wnt pathways were identified in our dataset. More than 50% of the non-canonical DEGs (11/19) were up-regulated while >50% of the canonical DEGs (16/27) were down-regulated in CabR cells (Fig 5A). Moreover, using the KEGG software, we determined that the Planar Cell Polarity and Calcium Wnt non-canonical pathways were broadly represented when compared to the canonical pathway in CabR cells (Fig 5B). Of note, in contrast to our Heat map analysis, the *cdc42 gene* surprisingly did not appeared within our KEGG analysis, as this gene is not part of the Wnt pathway genes list as defined by KEGG software (https://www.genome.jp/dbget-bin/www_bget?hsa04310). Accordingly, β-catenin protein was absent in CabR cells while highly up-regulated in DocR suggesting an inhibition of the Wnt canonical pathway (Fig 6). Conversely, Ror2 protein but not its related Ror1 member was dramatically increased in CabR but not in DocR cells suggestive of a switch to the Wnt non-canonical pathway (Fig 6A). Interestingly, Wnt5a, an identified ligand of Ror2, was also found increased in CabR.

### Inhibiting both the Wnt/planar cell polarity and Wnt/Ca^2+^ pathways in cabazitaxel resistant cells increased sensitivity to cabazitaxel

The Planar Cell Polarity and Calcium pathways have been widely described as downstream components of the Wnt non-canonical pathway. To test the involvement of these two sub-pathways in CabR, we thus used pharmacological inhibitors [Gö6983 (PKC), SP600125 (JNK), and KN93 (CamKII/IV)] that we tested alone or in combination, followed by taxanes on age-matched and taxane-resistant Du145 cells. We then measured the effects of each treatment condition on cell apoptosis (Fig 6B, 6C and 6D). When treated with inhibitors alone, no significant effects were observed in all the cell lines tested (S4 Fig). In age-matched cells, SP600125 was found protective against taxane as previously described [63] (Fig 6B, 6C and 6D). In all other combinations, inhibitors showed a minimal increase in apoptosis compared to taxane alone except for SP600125/Gö6983 and SP600125/Gö6983/KN93 combined with Cab (Fig 6B, 6C and 6D). In contrast, apoptosis was found increased by up to 8 fold in CabR cells in which both of the Wnt/PCP and Wnt/Ca^2+^ pathways were inhibited, compared to less than 2 fold in DocR cells (Fig 6C and 6D). Accordingly, the expression of PKC, CamKII/IV, and JNK1-3 proteins was found significantly up-regulated in CabR cells when compared to age-matched and DocR cells (Fig 6E) re-emphasizing the participation of the Wnt non-canonical pathway in CabR.

### Role of the tyrosine kinase Ror2 receptor in cabazitaxel resistance

**Fig 7:**
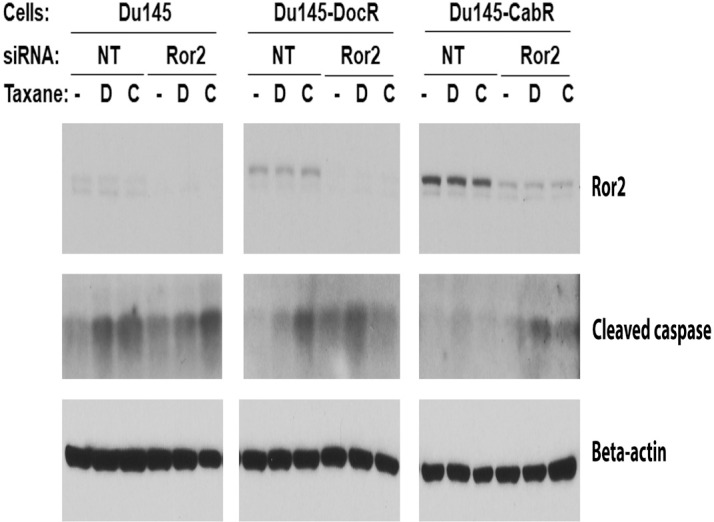
Ror2 knock-down resensitizes Du145-CabR cells to taxanes. Expression levels of Ror2 protein in association with cleaved caspase-3 in Du145 age-matched and taxane resistant cell lines transfected with non-targeting (NT) or Ror2-specific siRNA, and subsequently treated with Doc (D), Cab (C) or placebo. Beta-actin was used to verify protein equal loading.

**Fig 8:**
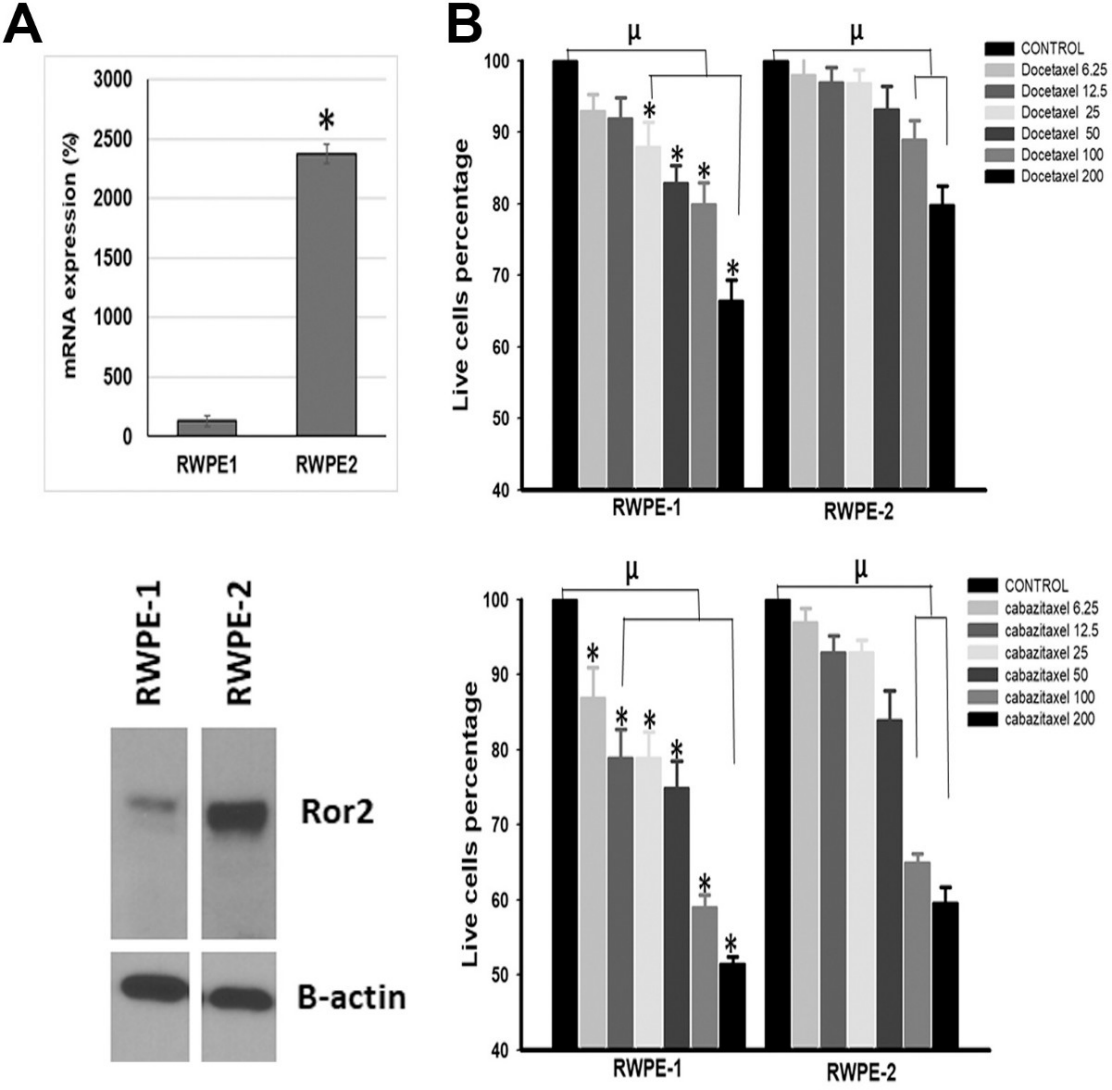
Effect of Ror2 expression levels on taxanes sensitivity in RWPE-1 and RWPE-2 prostatic epithelial cell lines. **(A)** mRNA (Top) and protein (Bottom) expression level for Ror2 in RPWE-1 and RWPE-2 cells quantified respectively by qRT-PCR and normalized to S15 mRNA (Top) and by western blotting using beta-actin as loading control (Bottom). **(B)** Growth curves of RWPE-1 and RWPE-2 cells in the presence of increasing concentrations of Doc and Cab. For the quantification data, the cells were fixed in 2% glutaraldehyde and stained with crystal violet then the optical density at 590 nm was measured using the Quad4 monochromators plate reader (TECAN Tecan Infinite M1000 Pro multi detection). Kruskal Wallis ANOVA followed by Dunn’s test was used to determine the significance of each dose compared to their respective control. Man-Whitney was used to compare Doc and Cab doses between RWPE-1 and RWPE-2. The symbol “μ” indicated that there is a significant difference between the dose in comparison to their respective control (p<0.001) and, the symbol ‘*’ indicated that there is a significant difference between RWPE-1 and RWPE-2 doses (p<0.05). The graphs represent the mean ± SEM of three different experiments.

To explore the role of the Ror2 tyrosine kinase receptor in CabR, we knocked-down the expression of Ror2 in Du145 parental and resistant cells. Cells were then treated with taxanes or placebo and assessed for cleaved caspase-3 expression as an indicator of apoptosis. As expected, we found that both Doc and Cab were able to induce the apoptosis of Du145 parental cells (Fig 7). In contrast, Ror2 knock-down in Du145-DocR slightly increase the cell sensitivity to Doc. Surprisingly, Ror2 knock-down reverted both DocR and CabR in Du145-CabR cells. To expand our knowledge to additional cell lines, we measured the expression level of Ror2 protein in several normal/tumor prostatic cell lines and found that the two genetically-related RWPE-1 and -2 cell lines respectively expressed low versus high levels of Ror2 mRNA and protein (Fig 8A). To test the effect of Ror2 expression level on taxane sensitivity, we thus seeded each cell line and subsequently treated them with increasing concentrations of Doc or Cab. Our results showed that both cell lines were sensitive to the two drugs (Fig 8B). As we previously demonstrated for PCa cell lines, both cell lines were also more sensitive to Cab than Doc. Interestingly, RWPE-2 (High Ror2 expression) were more resistant to both taxanes when compared to RWPE-1 (Low Ror2 expression) again suggesting that while Ror2 protein seems to play a role in taxane resistance, other determinant(s) may be required to direct either DocR or CabR.

## Discussion

Drug resistance, either intrinsic or acquired, is the major cause of failure to chemotherapy in patients with malignant tumors. While DocR has been widely described, CabR remains fairly new [35] with few single factors such as the Retinoblastoma tumor suppressor [36] and the SLCO1B3 transporter [37] which have been linked to Cab sensitivity. Using CabR variants established from MCF7 breast cancer cells, Duran et al. demonstrated a decrease in BRCA1 protein expression and induction of EMT thus validating our results (Fig 3) and linking EMT to CabR in both breast and prostate cancer [35]. In contrast, alterations in the FKBP7 chaperone protein, class III tubulins and the ABCB1 multidrug resistance protein were found to be responsible for Doc/Cab cross-resistance [38–43]. Accordingly, Cab was found to be more active in ABCB1(+) cell models due to its reduced affinity for P-gp compared to Doc [64]. In contrast, ABCC4 decreased Doc but not Cab efficacy in PCa cells *in vitro* [65]. Our dataset validated SLCO1B3 gene deregulation and re-identified the cell proliferation MAPKinase/ERK pathway as previously demonstrated by Hongo et al. [44]. Surprisingly, we did not identify ABCB1 as a DEG. We believe several reasons could explain this discrepancy. First, the experimental cell model used, breast vs prostate cells, Du145 vs PC3 cells, may lead to different gene expression profiles. Second, the use of pre-existing DocR cells to establish double resistant DocR/CabR cells could certainly play a role. Third, the dose as well as the schedule/regimen used to develop CabR cell lines may also explain this divergence. In two consecutive studies, Ramachandran et al. looked at the link between taxane sensitivity and DNA methylation. In the first study, the authors showed that growth arrest and DNA damage inducible-alpha (GADD45a), a pro-apoptotic gene, is frequently inactivated by methylation in PCa and contributes to Doc sensitivity [66]. In a follow-up study looking at Du145 Doc/Cab double resistant cells, the authors then showed that pre-treatment with the methyl-transferase inhibitor 5-azacytidine enhanced sensitivity to Cab in these cells supporting an impact of taxane drugs on chromatin organization [67]. Accordingly, Hongo et al. revealed in a pathway analysis that Du145 CabR cells presented enhanced gene clusters of cell division and mitotic nuclear division [44]. Using the same cell line, the authors also found an enhancement of the MAPK/ERK signaling. Accordingly, we identified the MAPK pathway as one of the top signaling pathway in our KEGG analysis (Du145 cells). We found 637 genes involved in cellular growth and proliferation, differentially deregulated in our Du145 cabazitaxel resistant cells when compared to the age-matched control cells (IPA analysis). Differentially, Hongo et al. showed that in contrast to Du145 cells, PC3 CabR cells had enhancement of PI3K/AKT signaling emphasizing as we stated earlier on cell specificity [44]. Additionally, the CCL2-CCR2 axis was identified as a potential therapeutic target against CabR in Du145 cells [45]. A recent study demonstrated that adipose stroma cells induced EMT in PCa cell lines leading to chemoresistance to various drugs including Taxanes [68] suggestive of a key role of cell interactions in sensitivity to drugs. In our experimental model, we used Du145 cells that we have submitted to dose escalation of taxane to mimic the drug lethal dose used in patients. As well, we established single resistant cell lines (DocR or CabR) based on the fact that recent clinical trials suggested that Cab could be used as first line chemotherapy in patients with PCa [9]. The molecular mechanisms of resistance attributed only to Cab thus represent an important question that remains unexplored.

A limitation of our study is that, we were not able to develop resistance in other cell lines than Du145. The reason underlying this phenomenon are unclear. One possibility could be the dose of drug used as well as the schedule/regimen used to develop CabR cell lines. In the two published studies, using PC3 and Du145 cells as models [42,44], the authors used lower doses of Cab to develop resistant cell lines; between 1 and 3nM. In an effort to mimic the lethal dose used in patients, we chose higher doses, up to 300nM. It is then possible that at this elevated concentration, PC3 cells are not able to recover from the treatment whereas Du145 cells do recover. In contrast to PC3 cells (https://www.atcc.org/products/all/CRL-1435.aspx#characteristics), Du145 are positive for testosterone 5-alpha reductase [69]. Interestingly, the steroid-5 alpha-reductase gene has been showed to be highly expressed in triple negative breast cancer cells also characterized by their resistance to chemotherapy and unfavorable prognosis [70]. As well, P53 has been link to taxanes sensitivity. As an example, Kim et al. study showed that Doc treatment induced low, moderate, and high level of apoptosis in LNCaP (P53 wt), Du145 (P53 mutant) and PC3 (P53 Null), respectively [71]. Our acquired resistance cellular model showed otherwise all the expected characteristics of resistance. Using *in vitro* clone formation and apoptosis assays, we found that age-matched parental cells were sensitive to both drugs. DocR were only resistant to Doc, while CabR showed a partial cross-resistance. Then, we investigated gene expression profiling by using a RNA Sequencing approach. The major diseases and disorders functions were cancer, organismal injury and abnormalities, and gastrointestinal disease. The main molecular and cellular functions included cellular movement, growth and proliferation, cell morphology, and cellular development, validating furthermore our model. Finally, the pathway analyses revealed Wnt signaling as one of the top signaling activated in CabR cells.

The Wnt canonical/β-catenin dependent and non-canonical/β-catenin independent signaling pathways play crucial roles in development and disease by transducing signals from the Wnt ligands via a complex network of receptors and co-receptors [72]. Although there are considerable overlaps between these two pathways, it is well recognized that the canonical pathway regulates cell proliferation and differentiation while the non-canonical regulates cell polarity. Furthermore, the Wnt non-canonical signaling is composed of two sub-pathways; the Wnt/Ca^2+^ and the Planar-cell polarity pathways (PCP). The Wnt/Ca^2+^ pathway modulates the cytoskeleton structure via the phospholipase C (PLC), Ca^2+^/calmodulin dependent protein kinase (CaMK), protein kinase-C (PKC) and NFAT [72], and the PCP pathway regulates cell movement and polarity via RhoA, Rac and Jun N-terminal kinase (JNK) [73]. To characterize the Wnt signaling pathway associated with CabR, we first measured the expression of β-catenin protein. While highly expressed in DocR cells, β-catenin completely disappeared in CabR cells suggesting a switch from the canonical to the non-canonical pathway. Interestingly, Bordonaro et al. previously demonstrated similar switch in colon cancer cells [74]. Importantly, Wnt5a ligand and its specific tyrosine kinase Ror2 receptor (effector of the Wnt non-canonical pathway) were found partly responsible for this induction. Moreover, the Wnt non-canonical as well as the inhibition of the Wnt canonical pathway have both been implicated in the resistance to enzulatamide and androgen deprivation therapies in CRPCa [75,76]. Accordingly, we found that Ror2 protein, a highly conserved receptor of the Wnt non-canonical pathway, but not Ror1 was dramatically increased in CabR but not in DocR cells suggestive of a switch to the Wnt non-canonical pathway. Interestingly, Wnt5a identified ligand of Ror2 [77], and also PKC, JNK1-3, and CamKII/IV non-canonical effectors were all up-regulated in our CabR cells. Ror2 (receptors tyrosine kinase-like orphan receptor 2) belongs to the pseudo-kinase family and is known to regulate several cellular processes including cell division and migration via the activation of the Wnt-PCP and -Ca^2+^ pathways. It also plays an enhancing role in the polarization and elongation of primordial germ cells [78]. In cancers such as in melanoma, breast, and prostate tumors among others, elevation of the Ror2 protein has been linked to increased cell migration resulting to tumor progression and chemo-resistance [79,80].

While the Wnt non-canonical pathway seems to be associated in CabR, the Wnt-β-catenin pathway is involved in DocR because of β-catenin protein which is highly expressed in DocR cells. Interestingly, the level of expression of the E-cadherin was also highly expressed in DocR cells. It is well known that E-cadherin mediates cell-cell adhesion. However, it has also been observed that E-cadherin have other functions than cell-cell adhesion and may act as a gene transcriptional regulator [81]. The cadherin-β-catenin pool was shown to translocate into the nucleus upon Wnt signaling pathway activation [82] which corroborate our immunofluorescence findings. In Howard et al. study, the authors found that cadherin was required for Wnt/β-catenin pathway activation and suggest that E-cadherin could be a positive transcriptional regulator of the Wnt/β-catenin pathway [83]. However, in other study, it is the Wnt/β-catenin pathway that have been shown to regulate E-cadherin expression which suggests that there is a feedback loop between E-cadherin and Wnt/β-catenin signaling pathway [84]. In our study, E-cadherin over-expression suggest that E-cadherin may act as a positive regulator of Wnt/β-catenin pathway or it is Wnt/β-catenin pathway that up-regulates E-cadherin in the acquisition of the resistance to docetaxel. These findings warrant further investigations.

To validate the involvement of the Wnt non-canonical pathway in CabR, we inhibited PKC, CamK and JNK proteins as well as knocked-down Ror2. Inhibition of JNKs had a protective effect against taxanes as previously described (62). Apoptosis was increased in CabR cells in which both of the PCP and Wnt/Ca^2+^ pathways were suppressed, when compared to DocR cells. Surprisingly, Ror2 down-regulation decreased the sensitivity of Du145 cells to both Doc and Cab. Looking at the expression levels of Ror2 in several prostatic cell lines, we found that RWPE-1 and -2, two parented prostatic cell lines, expressed respectively low and high Ror2 protein levels. Using cell proliferation assays, we demonstrated that both cell lines were sensitive to the two drugs with a higher sensitivity to Cab. Furthermore, RWPE-2 (High Ror2 expression) were more resistant to both taxanes when compared to RWPE-1 (Low Ror2 expression) thus validating our knock-down study and suggesting that while Ror2 protein seems to play a role in taxane resistance, other determinant(s) may be required to direct specificity toward DocR or CabR. Interestingly, *Dishevelled 1 (DVL1)*, a key effector of both Wnt-canonical and non-canonical was identified in our RNA sequencing data set. Furthermore, DVL1 was found deregulated in response to 5-azacytidine treatment in PCa cells reinforcing a possible link between CabR and chromatin organization [67]. As well, Post-Translational Modifications (PTMs) of DVL protein have been shown to modify DVL’s cell localization and function [85,86]. Direct PTMs on Ror2 protein also remains a possibility. Ror2 has been previously identified as sulfonated, and sulfonation of serine and threonine has been involved in multiple functions including protein assembly and signal transduction [87].

In conclusion, our data clearly identified the Wnt non-canonical pathway as a novel signaling involved in CabR in PCa cells. Furthermore, we also identified the Ror2 Tyrosine kinase receptor as an important determinant in taxane resistance. Still the precise effectors that determine and fix the resistance toward Doc or Cab remain unclear thus warranting more investigations.

## Supporting information

S1 Fig Expression and subcellular localization of E-cadherin. Representative fluorescence images of E-cadherin are shown in Age-matched, DocR and CabR cells. The images were taken using a Nikon confocal microscopy with a 60x objective. The images were analyzed using NIS software. Note that E-cadherin staining is stronger in DocR cells versus age-matched parental cells and that the signal disappeared in CabR.
(TIF)

S2 Fig Legend of the PANTHER Classification graph as represented in Fig 4D. (TIF)

S3 Fig DEGs previously associated with docetaxel resistance but not associated with CabR in our RNA sequencing dataset. (TIF)

S4 Fig Apoptosis in age-matched and taxanes-resistant Du145 cells treated with either Gö6983 (PKC inhibitor, 1μM, 2hrs), SP600125 (JNK inhibitor, 30μM, 2hrs), or KN93 (CamKII/IV inhibitor, 10μM, 4hrs. Note the absence of apoptotic effect of any of the pharmacological inhibitors when delivered as a single treatment agent.
(TIF)

S1 Data (XLSX)

S1 Raw images (PDF)
