# Supplementary figures and images for: The Wnt non-canonical signaling modulates cabazitaxel sensitivity in prostate cancer cells
Source: PLoS One. 2020 Jun 2;15(6):e0234078. doi: 10.1371/journal.pone.0234078 (PMC7266300; doi:10.1371/journal.pone.0234078)

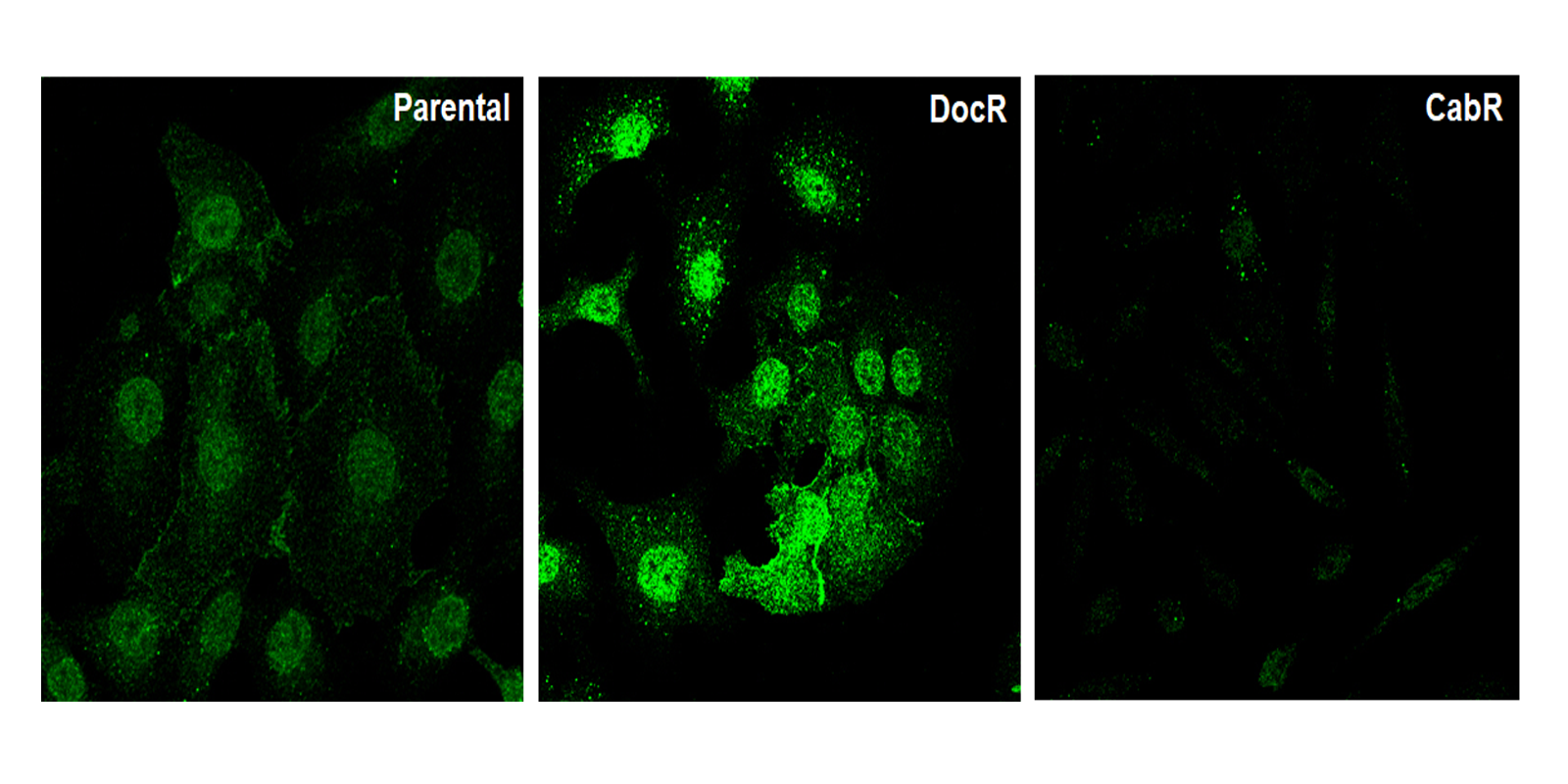

Supplement: S1 Fig — Representative fluorescence images of E-cadherin are shown in Age-matched, DocR and CabR cells. The images were taken using a Nikon confocal microscopy with a 60x objective. The images were analyzed using NIS software. Note that E-cadherin staining is stronger in DocR cells versus age-matched parental cells and that the signal disappeared in CabR. (TIF) [file pone.0234078.s001.tif]

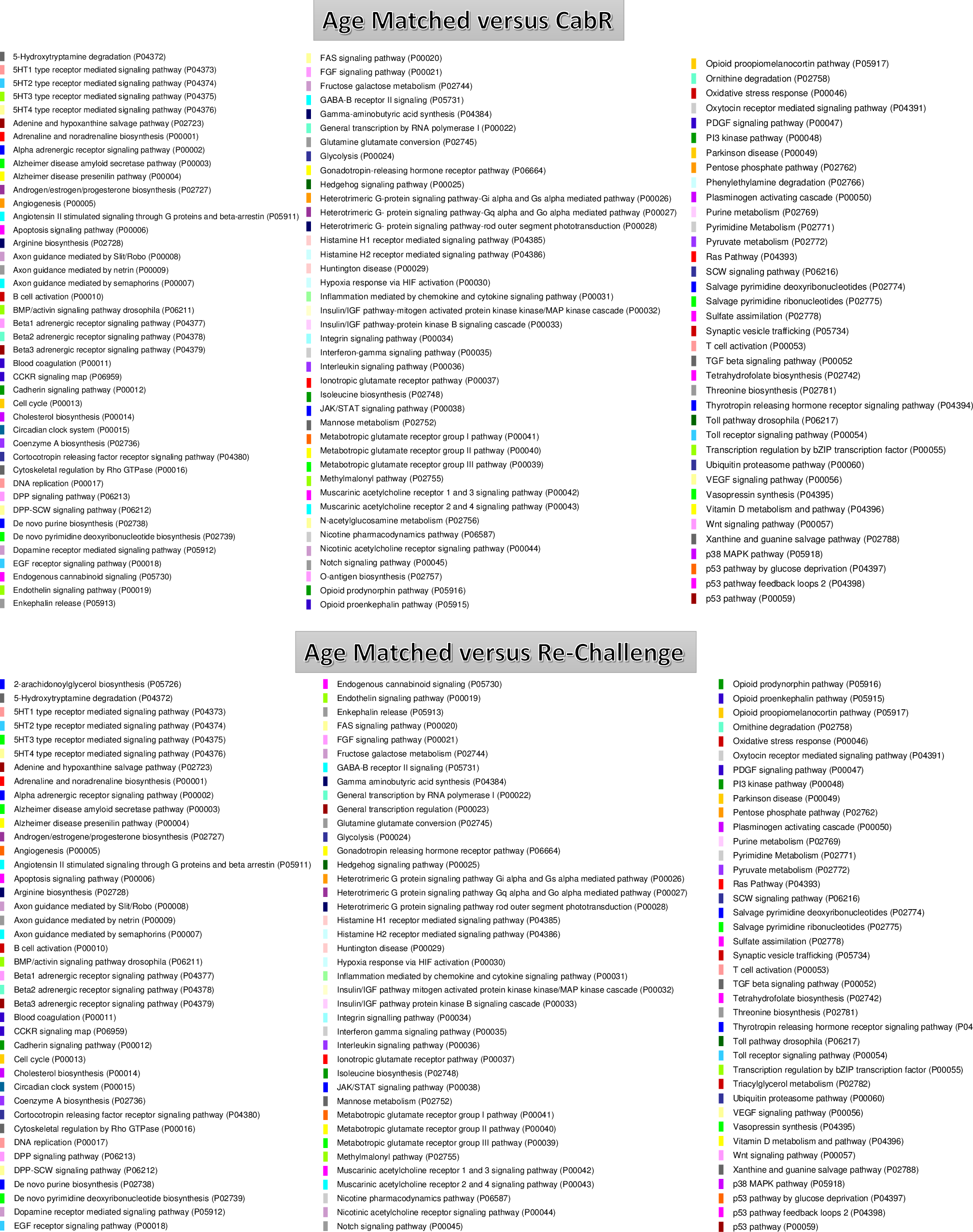

Supplement: S2 Fig — (TIF) [file pone.0234078.s002.tif]

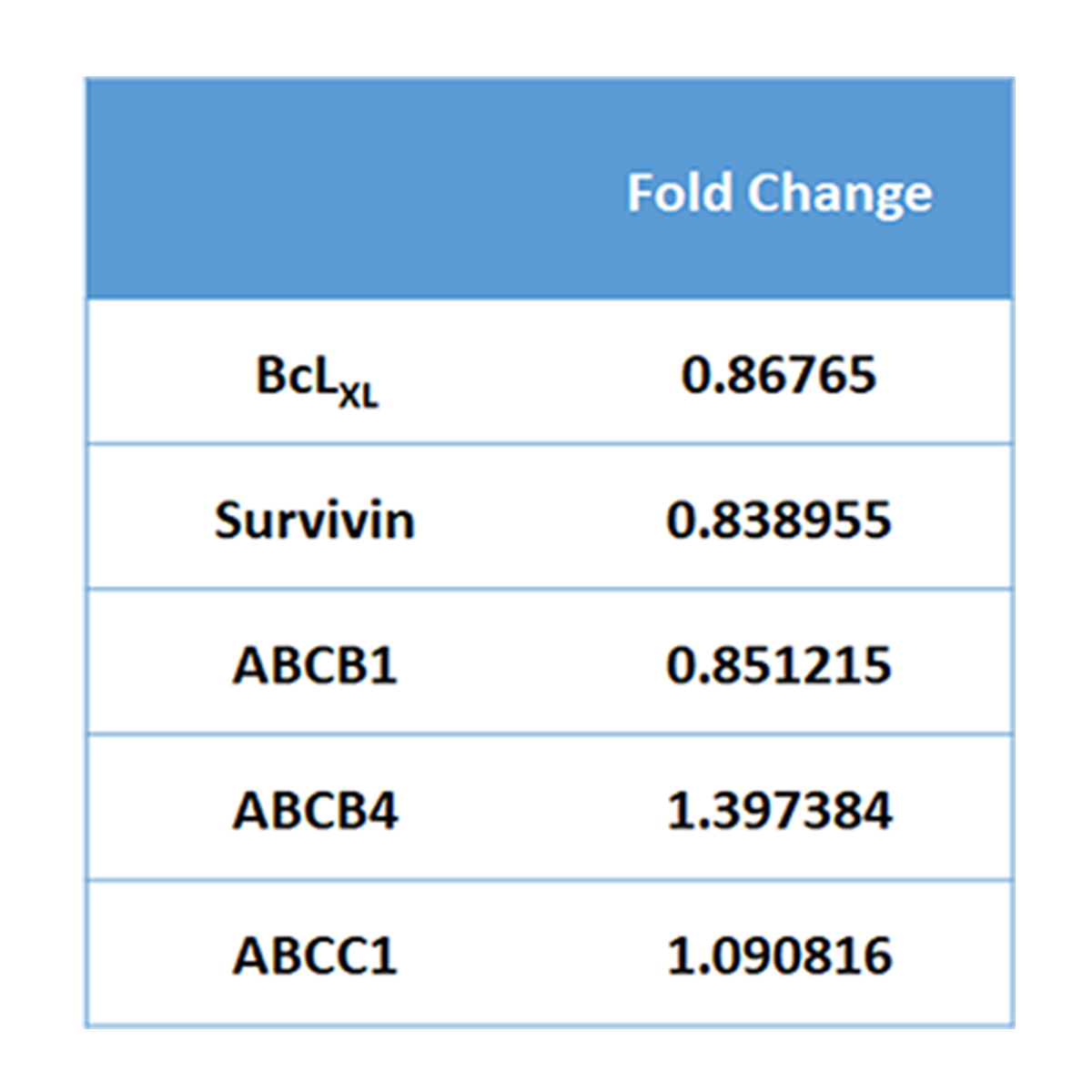

Supplement: S3 Fig — (TIF) [file pone.0234078.s003.tif]

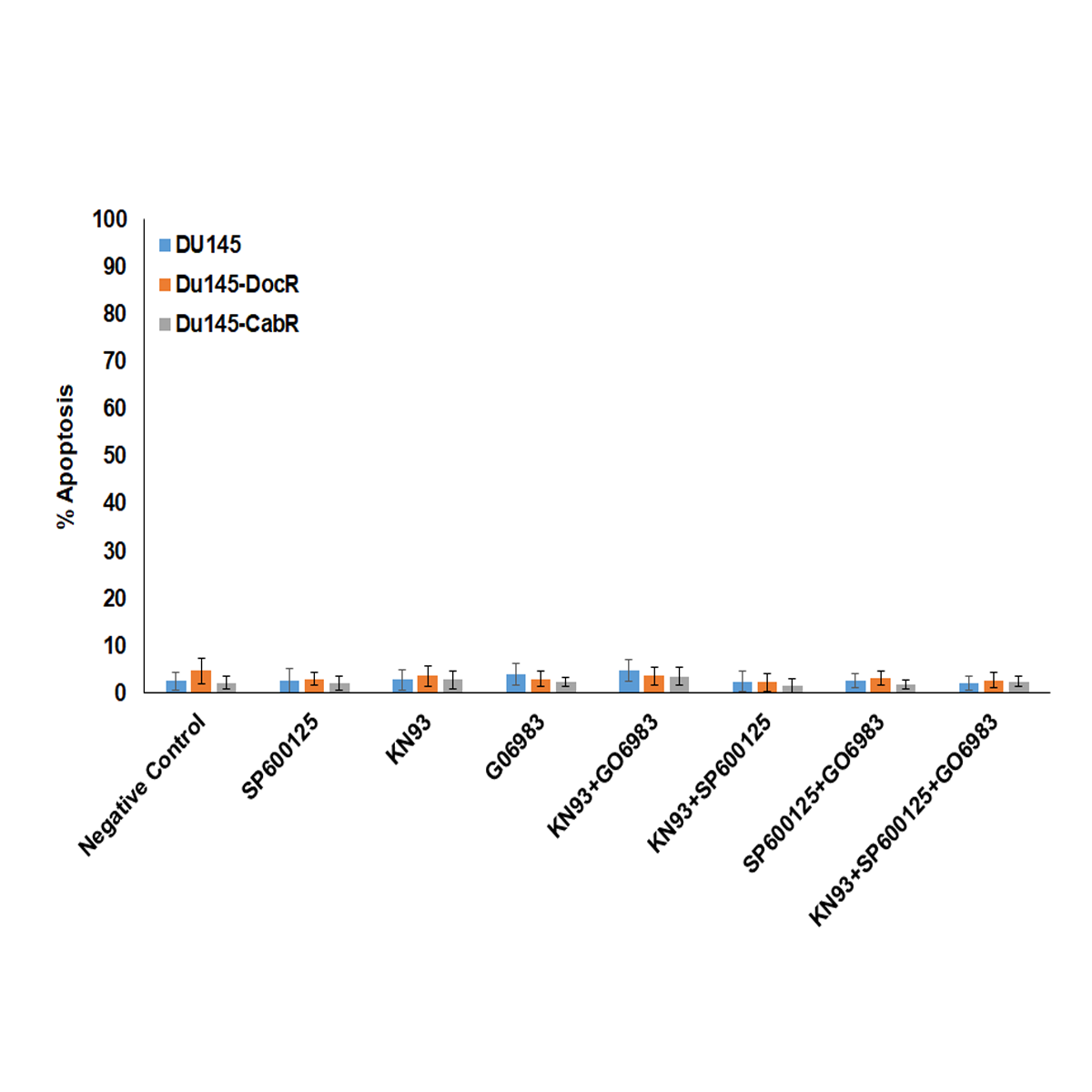

Supplement: S4 Fig — Note the absence of apoptotic effect of any of the pharmacological inhibitors when delivered as a single treatment agent. (TIF) [file pone.0234078.s004.tif]
