# Supplementary material for: The Wnt non-canonical signaling modulates cabazitaxel sensitivity in prostate cancer cells
Source: PLoS One. 2020 Jun 2;15(6):e0234078. doi: 10.1371/journal.pone.0234078 (PMC7266300; doi:10.1371/journal.pone.0234078)
Supplement: S1 Raw images — (PDF) [file pone.0234078.s006.pdf]

Cell Line Authentication Service

STR Profile Report

Sample Submitted By: Dr. Stephanie Filleur
Texas Tech Un. Health Sciences Center

Email Address: stephanie.filleur@ttuhsc.edu

ATCC Sales Order: SOJ24320

FTA Barcode: STRA3014

Cell Line Designation: Du 145 CabR

Date Sample Received: March 10, 2016

Report Date: March 16, 2016

Methodology: Seventeen short tandem repeat (STR) loci plus the gender determining locus, Amelogenin, were amplified using the commercially available PowerPlex® 18D Kit from Promega. The cell line sample was processed using the ABI Prism® 3500xl Genetic Analyzer. Data were analyzed using GeneMapper® ID-X v1.2 software (Applied Biosystems). Appropriate positive and negative controls were run and confirmed for each sample submitted.

Data Interpretation Cell lines were authenticated using Short Tandem Repeat (STR) analysis as described in 2012 in ANSI Standard (ASN-0002) *Authentication of Human Cell Lines: Standardization of STR Profiling* by the ATCC Standards Development Organization (SDO) and in Capes-Davis et al., Match criteria for human cell line authentication: Where do we draw the line? Int. J. Cancer. 2012 Nov 8. doi: 10.1002/ijc.27931

ATCC performs STR Profiling following ISO 9001:2008 and ISO/IEC 17025:2005 quality standards.

There are no warranties with respect to the services or results supplied, express or implied, including, without limitation, any implied warranty of merchantability or fitness for a particular purpose. Neither ATCC nor Promega is liable for any damages or injuries resulting from receipt and/or improper, inappropriate, negligent or other wrongful use of the test results supplied, and/or from misidentification, misrepresentation, or lack of accuracy of those results. Your exclusive remedy against ATCC, Promega and those supplying materials used in the services for any losses or damage of any kind whatsoever, whether in contract, tort, or otherwise, shall be, at Promega's option, refund of the fee paid for such service or repeat of the service.

The ATCC trademark and trade name, any and all ATCC catalog numbers are trademarks of the American Type Culture Collection. PowerPlex is a registered trademark of Promega Corporation. Applied Biosystems, ABI Prism and GeneMapper are registered trademarks of Life Technologies Corporation.

Technical questions?

ATCC Technical Support
(800) 638-6597 / +1 703-365-2700
STRTechSupport@atcc.org

Ordering questions?

800-638-6597 or 703-365-2700
Fax 703-365-2750
Email: STRtesting@atcc.org

Cell Line Authentication Service
STR Profile Report

FTA Barcode: STRA3014
ATCC Sales Order: SOJ24320

**Test Results for Submitted Sample**					**ATCC Reference Database Profile**			
**Loci**	**Query Profile: Du 145 CabR**				**Database Profile: DU 145**			
**D3S1358**	16							
**TH01**	7				7			
**D21S11**	30	33						
**D18S51**	12							
**Penta_E**	12	14						
**D5S818**	10	13			10	13		
**D13S317**	12	13	14		12	13	14	
**D7S820**	7	10	11		7	10	11	
**D16S539**	11	12	13		11	13		
**CSF1PO**	11				10	11		
**Penta_D**	9	13						
**Amelogenin**	X	Y			X	Y		
**vWA**	18				17	18	19	
**D8S1179**	13	14						
**TPOX**	10	11			11			
**FGA**	22	23						
**D19S433**	12	13						
**D2S1338**	16							

Number of shared alleles between query sample and database profile:	16
Total number of alleles in the database profile:	19
Percent match between the submitted sample and the database profile:	84

The allele match algorithm compares the 8 core loci plus amelogenin only, even though alleles from all loci will be reported when available.

NOTE: *Loci highlighted in grey (8 core STR loci plus Amelogenin) can be made public to verify cell identity. In order to protect the identity of the donor,* ***please do not publish*** *the allele calls from all the STR loci tested. Electropherograms showing raw data are attached.*

Explanation of Test Results

Cell lines with ≥ 80% match are considered to be related; i.e., derived from a common ancestry. Cell lines with between a 55% to 80% match require further profiling for authentication of relatedness.

- [ ] The submitted sample profile is human, but not a match for any profile in the ATCC STR database
- [ ] The submitted profile is an exact match for the following ATCC human cell line(s) in the ATCC STR database (8 core loci plus Amelogenin):
- [X] The submitted profile is similar to the following ATCC human cell line(s):
  HTB-81 (DU 145)

Additional Comments:
Submitted sample, STRA3014 (Du 145 CabR) is similar to ATCC cell line HTB-81 (DU 145).

e-Signature, Technician:	snicholson 03/16/16
e-Signature, Reviewer:	Kkindig 03/16/16

Cell Line Authentication Service
STR Profile Report

FTA Barcode: STRA3014
ATCC Sales Order: SOJ24320

Addendum: Comparative Output from the ATCC STR Profile Database

% Match	ATCC® Cat. No.	Designation	D5S818	D13S317	D7S820	D16S539	vWA	THO1	AMEL	TPOX	CSF1PO
100	N/A	Du 145 CabR	10,13	12,13,14	7,10,11	11,12,13	18	7	X,Y	10,11	11
84	HTB-81	DU 145	10,13	12,13,14	7,10,11	11,13	17,18,19	7	X,Y	11	10,11

Definitions of terms used in this report:

Peak Area Difference (PAD):
Refers to a heterozygous peak imbalance.
Two alleles at a single locus should amplify in a similar manner; and therefore produce peaks of similar height and area. Peaks which are above threshold (50 rfu) but are not of similar area, within 50% of each other, are referred to as a PAD. Due to their nature cell lines do not amplify in the same manner as a sample taken from a fresh buccal swab. PAD is far more common in cell line samples.

Stutter:
A stutter peak is a small peak which occurs immediately before the true peak. It is defined as being a single repeat unit smaller than the true peak. The stutter peak should be less than 15% of the true peak. The stutter is caused by the polymerase.

+4 Peak:
A +4 is similar to a stutter but occurs immediately after the true peak. A stutter peak should be less than 5% for a homozygous and 10% for a heterozygous.

Below Threshold Peak(s):
Cell lines can produce unusual profiles and occasionally a peak will amplify poorly and be below threshold. Where we find a below threshold peak which we believe is valid we indicate it as a below threshold peak. Our cell line analysis criteria, Homozygous and Heterozygous peaks must be equal to or above the set height threshold for it to be considered a true peak.

Ladder/ Off Ladder Peak(s):
The allelic ladder consists of most or all known alleles in the population and allows for precise assignment of alleles. Those which do not align are termed ‘off ladder.

Artifact:
A non-allelic product of the amplification process, an anomaly of the detection process, or a by-product of primer synthesis

Pull-up:
A term used to describe when signal from one dye color channel produces artificial peaks in another, usually adjacent, color.

Spike:
An extraneous peak resulting from dust, dried polymer, an air bubble, or an electrical surge.

Dye blob:
Free dye not coupled to primer that can be injected into the capillary (A known and documented dye blob is often found at the D3S1358 locus.)

Sample Name	SQ
STRA3014_Du-145-CabR_External_Sample_y	

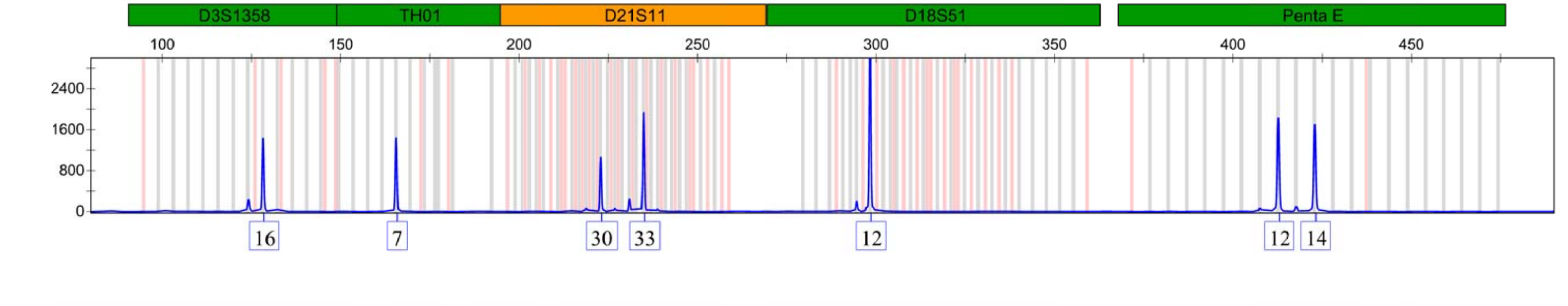

STRA3014_Du-145-CabR_External_Sample_y

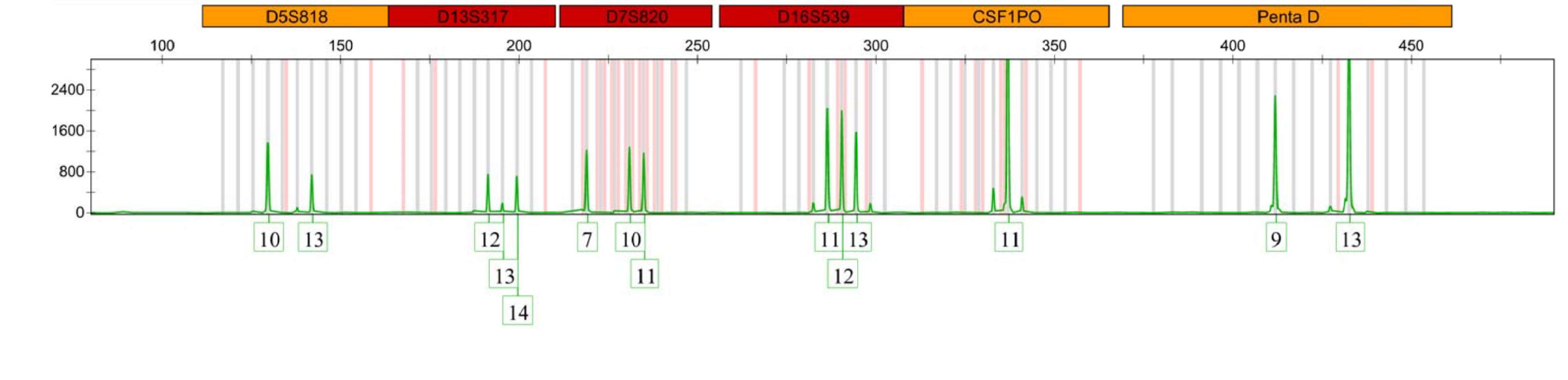

Sample Name	SQ
STRA3014_Du-145-CabR_External_Sample_y	

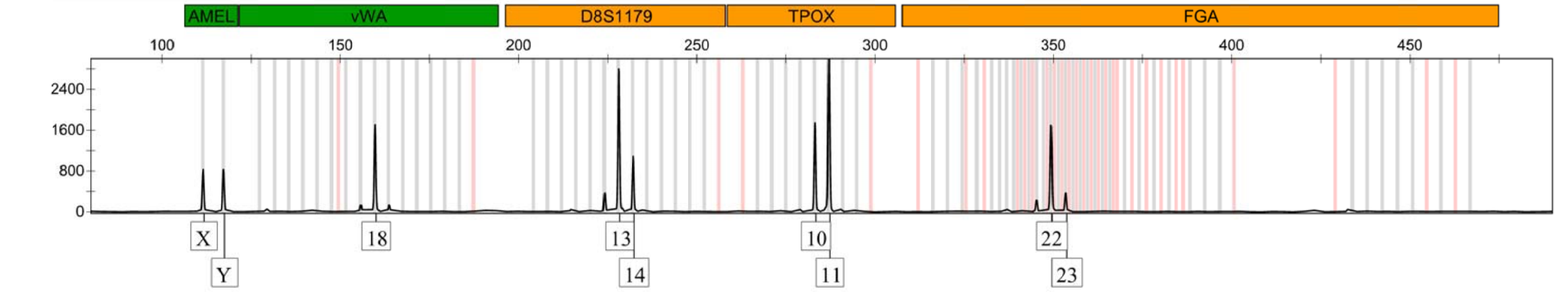

STRA3014_Du-145-CabR_External_Sample_y

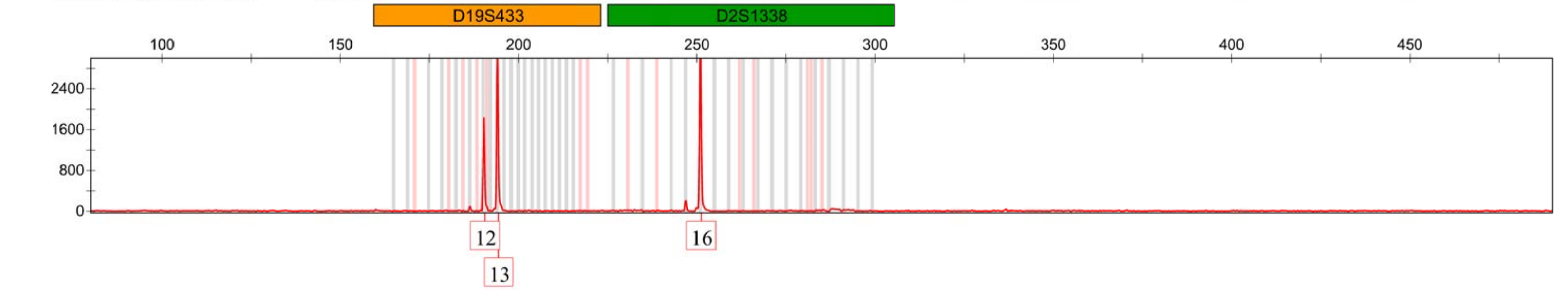

Cell Line Authentication Service

STR Profile Report

Sample Submitted By: Stephanie Filleur
Texas Tech Un. Health Sciences Center

Email Address: stephanie.filleur@ttuhsc.edu

ATCC Sales Order: SOJ24320

FTA Barcode: STRA3015

Cell Line Designation: Du 145

Date Sample Received: March 10, 2016

Report Date: March 16, 2016

Methodology: Seventeen short tandem repeat (STR) loci plus the gender determining locus, Amelogenin, were amplified using the commercially available PowerPlex® 18D Kit from Promega. The cell line sample was processed using the ABI Prism® 3500xl Genetic Analyzer. Data were analyzed using GeneMapper® ID-X v1.2 software (Applied Biosystems). Appropriate positive and negative controls were run and confirmed for each sample submitted.

Data Interpretation Cell lines were authenticated using Short Tandem Repeat (STR) analysis as described in 2012 in ANSI Standard (ASN-0002) *Authentication of Human Cell Lines: Standardization of STR Profiling* by the ATCC Standards Development Organization (SDO) and in Capes-Davis et al., Match criteria for human cell line authentication: Where do we draw the line? Int. J. Cancer. 2012 Nov 8. doi: 10.1002/ijc.27931

ATCC performs STR Profiling following ISO 9001:2008 and ISO/IEC 17025:2005 quality standards.

There are no warranties with respect to the services or results supplied, express or implied, including, without limitation, any implied warranty of merchantability or fitness for a particular purpose. Neither ATCC nor Promega is liable for any damages or injuries resulting from receipt and/or improper, inappropriate, negligent or other wrongful use of the test results supplied, and/or from misidentification, misrepresentation, or lack of accuracy of those results. Your exclusive remedy against ATCC, Promega and those supplying materials used in the services for any losses or damage of any kind whatsoever, whether in contract, tort, or otherwise, shall be, at Promega's option, refund of the fee paid for such service or repeat of the service.

The ATCC trademark and trade name, any and all ATCC catalog numbers are trademarks of the American Type Culture Collection. PowerPlex is a registered trademark of Promega Corporation. Applied Biosystems, ABI Prism and GeneMapper are registered trademarks of Life Technologies Corporation.

Technical questions?

ATCC Technical Support
(800) 638-6597 / +1 703-365-2700
STRTechSupport@atcc.org

Ordering questions?

800-638-6597 or 703-365-2700
Fax 703-365-2750
Email: STRtesting@atcc.org

Cell Line Authentication Service
STR Profile Report

FTA Barcode: STRA3015
ATCC Sales Order: SOJ24320

**Test Results for Submitted Sample**					**ATCC Reference Database Profile**			
**Loci**	**Query Profile: Du 145**				**Database Profile: DU 145**			
**D3S1358**	16							
**TH01**	7				7			
**D21S11**	30	33						
**D18S51**	12							
**Penta_E**	12	14						
**D5S818**	10	13	14		10	13		
**D13S317**	12	13	14		12	13	14	
**D7S820**	7	10	11		7	10	11	
**D16S539**	11	13			11	13		
**CSF1PO**	10	11			10	11		
**Penta_D**	9	13						
**Amelogenin**	X	Y			X	Y		
**vWA**	17	18			17	18	19	
**D8S1179**	13	14						
**TPOX**	11				11			
**FGA**	22							
**D19S433**	13							
**D2S1338**	16	17						

Number of shared alleles between query sample and database profile:	18
Total number of alleles in the database profile:	19
Percent match between the submitted sample and the database profile:	95

The allele match algorithm compares the 8 core loci plus amelogenin only, even though alleles from all loci will be reported when available.

NOTE: *Loci highlighted in grey (8 core STR loci plus Amelogenin) can be made public to verify cell identity. In order to protect the identity of the donor,* ***please do not publish*** *the allele calls from all the STR loci tested. Electropherograms showing raw data are attached.*

Explanation of Test Results

Cell lines with ≥ 80% match are considered to be related; i.e., derived from a common ancestry. Cell lines with between a 55% to 80% match require further profiling for authentication of relatedness.

☐ The submitted sample profile is human, but not a match for any profile in the ATCC STR database

☐ The submitted profile is an exact match for the following ATCC human cell line(s) in the ATCC STR database (8 core loci plus Amelogenin):

☒ The submitted profile is similar to the following ATCC human cell line(s):
HTB-81 (DU 145)

Additional Comments:
Submitted sample, STRA3015 (Du 145) is similar to ATCC cell line HTB-81 (DU 145).

e-Signature, Technician:	snicholson 03/16/16
e-Signature, Reviewer:	Kkindig 03/16/16

Cell Line Authentication Service
STR Profile Report

FTA Barcode: STRA3015
ATCC Sales Order: SOJ24320

Addendum: Comparative Output from the ATCC STR Profile Database

% Match	ATCC® Cat. No.	Designation	D5S818	D13S317	D7S820	D16S539	vWA	THO1	AMEL	TPOX	CSF1PO
100	N/A	Du 145	10,13,14	12,13,14	7,10,11	11,13	17,18	7	X,Y	11	10,11
95	HTB-81	DU 145	10,13	12,13,14	7,10,11	11,13	17,18,19	7	X,Y	11	10,11

Definitions of terms used in this report:

Peak Area Difference (PAD):
Refers to a heterozygous peak imbalance.
Two alleles at a single locus should amplify in a similar manner; and therefore produce peaks of similar height and area. Peaks which are above threshold (50 rfu) but are not of similar area, within 50% of each other, are referred to as a PAD. Due to their nature cell lines do not amplify in the same manner as a sample taken from a fresh buccal swab. PAD is far more common in cell line samples.

Stutter:
A stutter peak is a small peak which occurs immediately before the true peak. It is defined as being a single repeat unit smaller than the true peak. The stutter peak should be less than 15% of the true peak. The stutter is caused by the polymerase.

+4 Peak:
A +4 is similar to a stutter but occurs immediately after the true peak. A stutter peak should be less than 5% for a homozygous and 10% for a heterozygous.

Below Threshold Peak(s):
Cell lines can produce unusual profiles and occasionally a peak will amplify poorly and be below threshold. Where we find a below threshold peak which we believe is valid we indicate it as a below threshold peak. Our cell line analysis criteria, Homozygous and Heterozygous peaks must be equal to or above the set height threshold for it to be considered a true peak.

Ladder/ Off Ladder Peak(s):
The allelic ladder consists of most or all known alleles in the population and allows for precise assignment of alleles. Those which do not align are termed ‘off ladder.

Artifact:
A non-allelic product of the amplification process, an anomaly of the detection process, or a by-product of primer synthesis

Pull-up:
A term used to describe when signal from one dye color channel produces artificial peaks in another, usually adjacent, color.

Spike:
An extraneous peak resulting from dust, dried polymer, an air bubble, or an electrical surge.

Dye blob:
Free dye not coupled to primer that can be injected into the capillary (A known and documented dye blob is often found at the D3S1358 locus.)

Sample Name	SQ
STRA3015_Du-145_External_Sample_v	

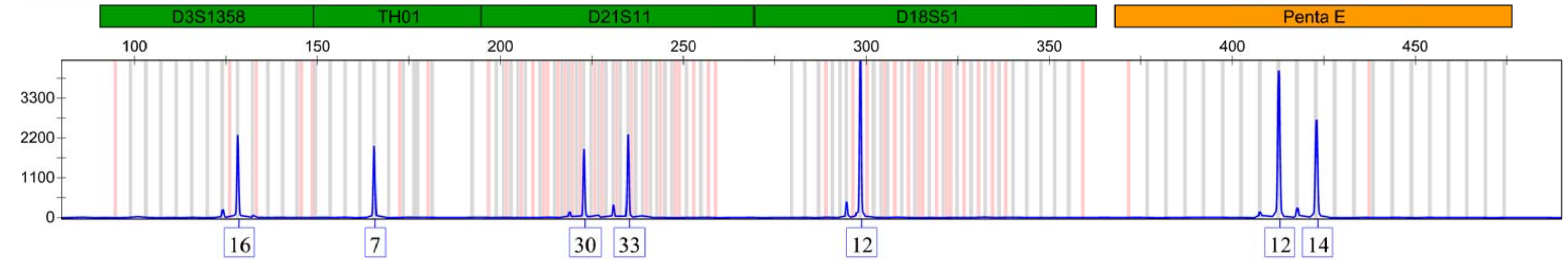

STRA3015_Du-145_External_Sample_v

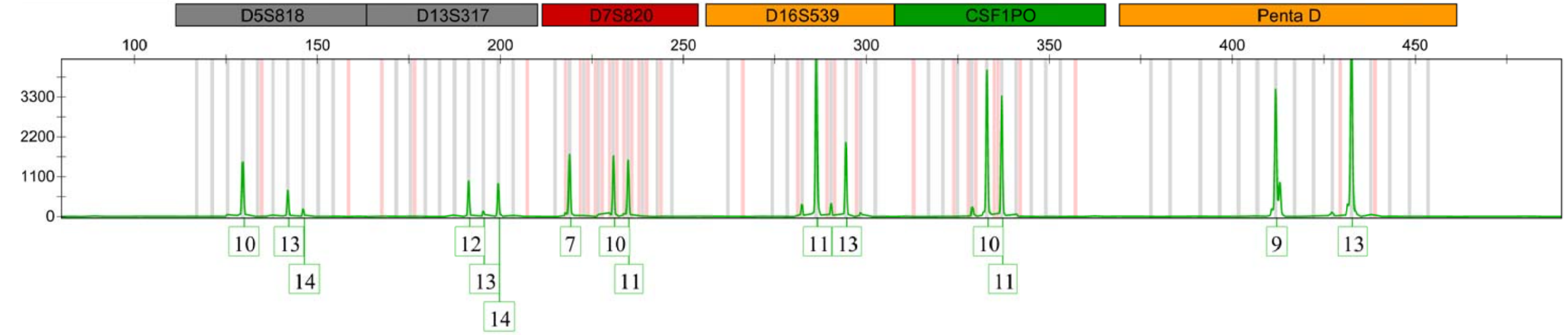

Sample Name	SQ
STRA3015_Du-145_External_Sample_y	

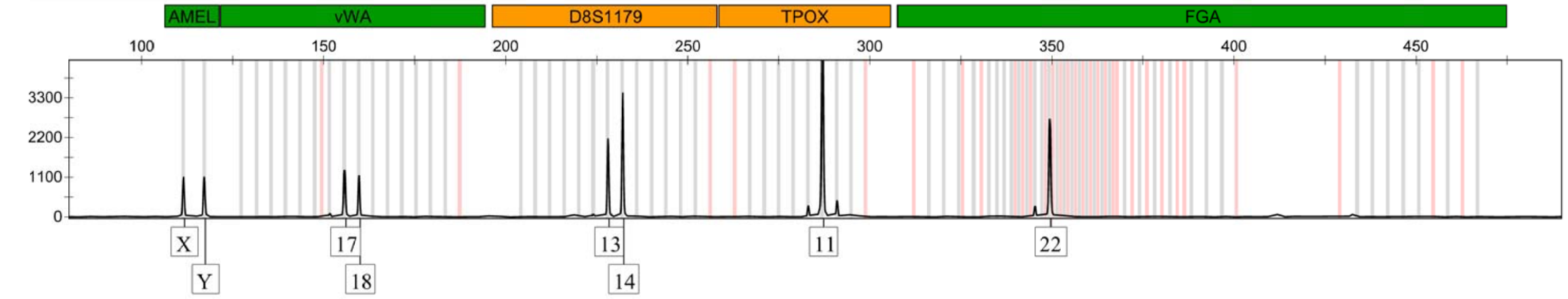

STRA3015_Du-145_External_Sample_y

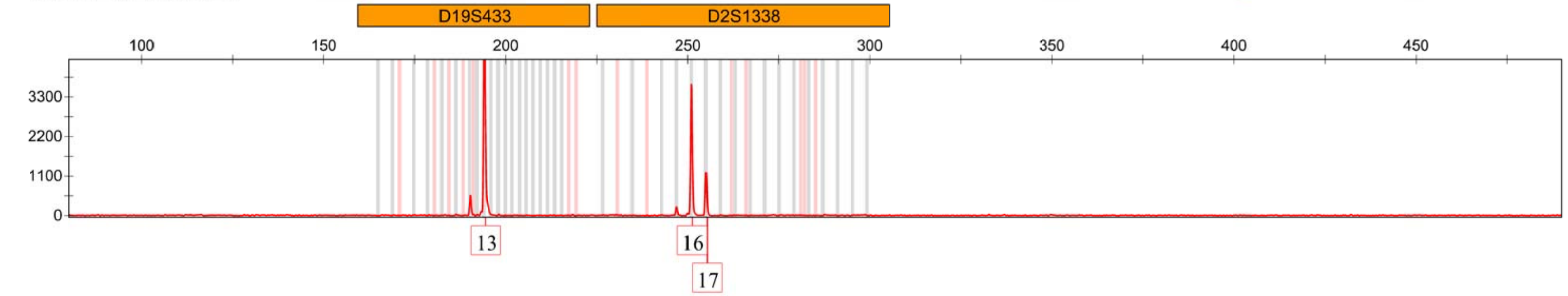

Fig3D

1- Du145 (07/14)
2- Du145-DocR (07/14)
3- Du145-CabR (07/14)
4- Du145 (07/22)
5-Du145-DocR (07/22)
6-Du145-CabR (07/22)
7- Du145 (07/28)
8- Du145-DocR (07/28)
9- Du145-CabR (07/28)

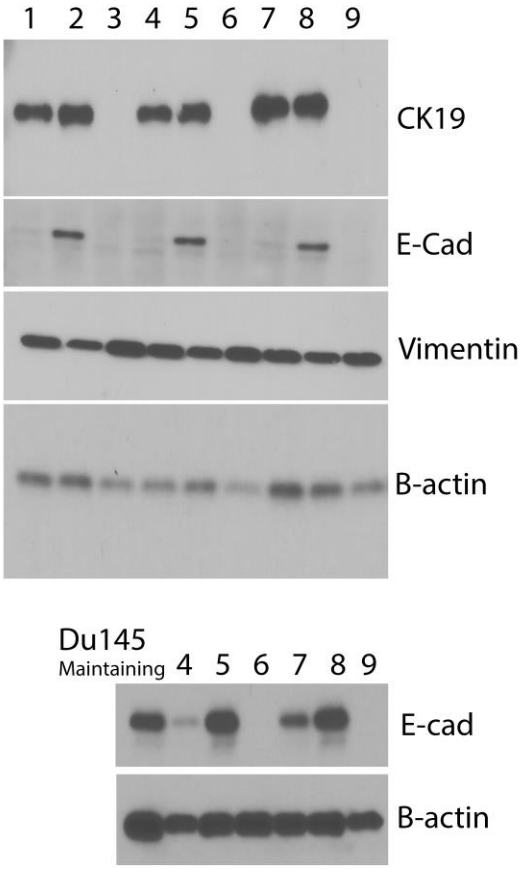

Fig6A

1- Du145 (07/14)
2- Du145-DocR (07/14)
3- Du145-CabR (07/14)
4- Du145 (07/22)
5-Du145-DocR (07/22)
6-Du145-CabR (07/22)
7- Du145 (07/28)
8- Du145-DocR (07/28)
9- Du145-CabR (07/28)

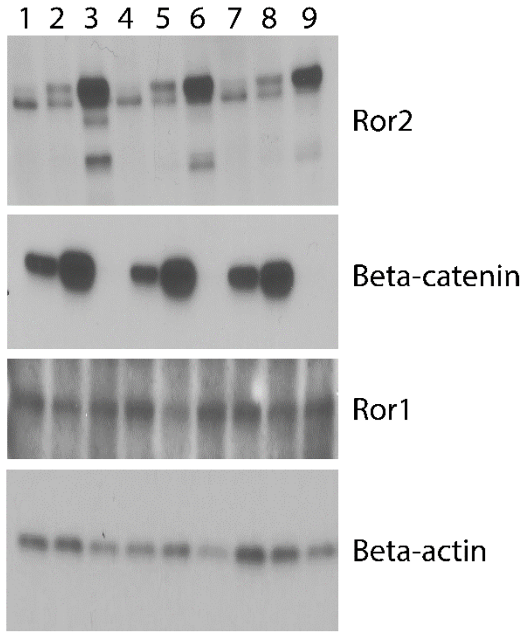

Fig6E

1- Du145
2- Du145-DocR
3- Du145-DocR
4- Du145-CabR
5- Du145-CabR

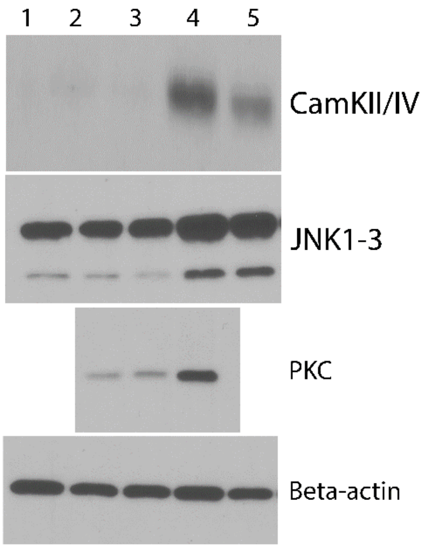

6- Du145 07/22
7- Du145-DocR 07/22
8- Du145-CabR 07/22

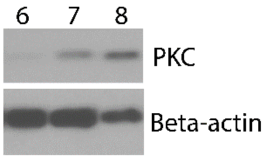

Fig 7

1- Du145 / NT / -
2- Du145 / NT / Doc
3- Du145 / NT / Cab
4- Du145 / Ror / -
5- Du145 / Ror / Doc
6- Du145 / Ror / Cab
7- Du145-DocR / NT / -
8- Du145-DocR / NT / Doc
9- Du145-DocR / NT / Cab
10- Du145-DocR / Ror / -
11- Du145-DocR / Ror / Doc
12- Du145-DocR / Ror / Cab
13- Du145-CabR / NT / -
14- Du145-CabR / NT / Doc
15- Du145- CabR / NT / Cab
16- Du145-CabR / Ror / -
17- Du145-CabR / Ror / Doc
18- Du145-CabR / Ror / Cab

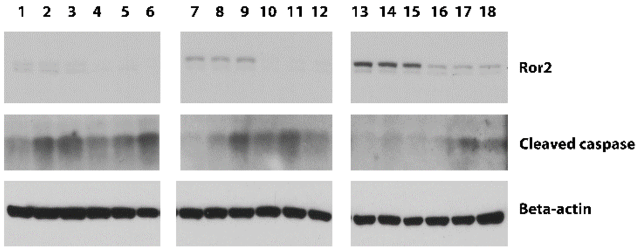

Fig8A

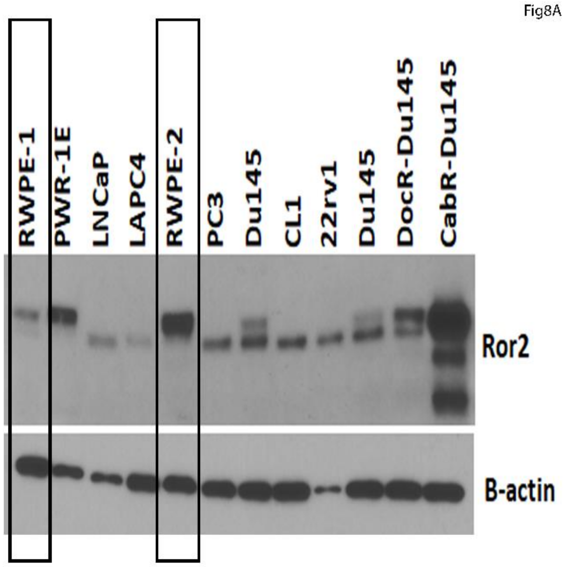
RWPE-1
PWR-1E
LNCaP
LAPC4
RWPE-2
PC3
Du145
CL1
22rv1
Du145
DocR-Du145
CabR-Du145
Ror2
B-actin
